# Testing efficacy of distance and tree-based methods for DNA barcoding of grasses (Poaceae tribe *Poeae*) in Australia

**DOI:** 10.1371/journal.pone.0186259

**Published:** 2017-10-30

**Authors:** Joanne L. Birch, Neville G. Walsh, David J. Cantrill, Gareth D. Holmes, Daniel J. Murphy

**Affiliations:** Royal Botanic Gardens Victoria, Melbourne, Victoria, Australia; Istituto di Biologia e Biotecnologia Agraria Consiglio Nazionale delle Ricerche, ITALY

## Abstract

In Australia, Poaceae tribe *Poeae* are represented by 19 genera and 99 species, including economically and environmentally important native and introduced pasture grasses [e.g. *Poa* (Tussock-grasses) and *Lolium* (Ryegrasses)]. We used this tribe, which are well characterised in regards to morphological diversity and evolutionary relationships, to test the efficacy of DNA barcoding methods. A reference library was generated that included 93.9% of species in Australia (408 individuals, $\bar{x}$ = 3.7 individuals per species). Molecular data were generated for official plant barcoding markers (*rbc*L, *mat*K) and the nuclear ribosomal internal transcribed spacer (ITS) region. We investigated accuracy of specimen identifications using distance- (nearest neighbour, best-close match, and threshold identification) and tree-based (maximum likelihood, Bayesian inference) methods and applied species discovery methods (automatic barcode gap discovery, Poisson tree processes) based on molecular data to assess congruence with recognised species. Across all methods, success rate for specimen identification of genera was high (87.5–99.5%) and of species was low (25.6–44.6%). Distance- and tree-based methods were equally ineffective in providing accurate identifications for specimens to species rank (26.1–44.6% and 25.6–31.3%, respectively). The ITS marker achieved the highest success rate for specimen identification at both generic and species ranks across the majority of methods. For distance-based analyses the best-close match method provided the greatest accuracy for identification of individuals with a high percentage of “correct” (97.6%) and a low percentage of “incorrect” (0.3%) generic identifications, based on the ITS marker. For tribe *Poeae*, and likely for other grass lineages, sequence data in the standard DNA barcode markers are not variable enough for accurate identification of specimens to species rank. For recently diverged grass species similar challenges are encountered in the application of genetic and morphological data to species delimitations, with taxonomic signal limited by extensive infra-specific variation and shared polymorphisms among species in both data types.

## Introduction

Poaceae tribe *Poeae* R.Br. are some of the most economically and environmentally important temperate native and introduced pasture and turf grasses, including *Lolium* L. (Ryegrasses), *Poa* L. (Tussock-grasses), and *Puccinellia* Parl. (Salt-grasses) [1,2]. These taxa provide a wide range of agricultural and ecological services such as forage, wildlife habitat, and restoration of salinized soils and are the focus of intensive management efforts in both agricultural and conservation contexts. Poaceae tribe *Poeae* include 19 genera and 99 species in Australia [2]. Species identifications typically rely on inflorescence, spikelet or floret features. However, where samples lack good flowering and/or fruiting material, identification of tribe *Poeae* taxa remains challenging or impossible. Demand remains high for a molecular barcoding methodology that can be used to accurately identify grass taxa.

DNA barcoding uses short DNA sequences, typically from a standard marker or markers, which may be used to address two distinct goals: firstly, to facilitate identification of unknown specimens and secondly, to guide species discovery. For plants, the “official” DNA barcodes are the plastid *rbc*L and *mat*K markers [3]. As a result of the low levels of variability within these markers [4], additional markers (for example, *psb*A-*trn*H [5–8], ribosomal internal transcribed spacer (ITS) [9–11], and *rpL*32-*trn*L [12]) and combinations of markers [13] have been applied in efforts to increase resolution for determination of specimens.

An accurate DNA barcoding methodology for grasses could provide significant benefits [14], ranging from determination of fragmentary and infertile plant samples [15], herbivore diet analyses (e.g. [16–18]), and studies of community structure [19]. However, the accuracy and limitations of DNA barcoding techniques for assignment of taxonomic identify have not been comprehensively tested for grasses. In a study of Australian grasses, Syme *et al*. [15] concluded that ITS provided better accuracy than *rbc*L and *mat*K for identification of unknown subf. *Pooideae* tribe *Stipeae* grass individuals. In an investigation of six candidate DNA barcode markers, Wang *et al*. [20] concluded that *mat*K and *atp*F had the highest success rates for identification of five exotic grass species present in Australia. Saarela *et al*. [21] included three tribe *Poeae* genera (*Festuca*, *Poa*, and *Puccinellia*) for DNA barcoding of the Canadian arctic flora and found that 30% of the *Festuca* species and 46% of the *Poa* species sampled could be distinguished using *rbc*L plus *mat*K. Peterson *et al*. [12] concluded that ITS holds the greatest discriminatory power (96.0% of taxa) followed by *rpl*32-*trn*L (25.6% of taxa) for identification of unknown North American subf. *Chloridoideae* species.

A range of methods are available, that apply different criteria for the purposes of assigning taxonomic identity based on DNA sequence data (for an overview of these methods see, for example [22,23]). However, few studies have tested and compared the accuracy among a range of distance- and tree-based DNA barcoding methods to assess their relative accuracy for plant lineages [15], relying instead on standard distance-based (e.g. quantification of genetic distance or neighbour joining dendrogram construction) or tree-based (e.g. application of maximum likelihood or Bayesian criteria for phylogeny reconstruction) methods.

New methods that potentially increase the accuracy of DNA barcoding methods have not yet been applied to the majority of plant lineages, including grasses. For example, estimation and application of a pairwise genetic distance threshold that distinguishes inter- and intra-specific comparisons can increase accuracy of DNA barcoding methods by preventing misidentifications due to the absence of congeneric or conspecific individuals in the reference library [24]. With the exception of a small number of studies that have applied threshold distances (e.g. [7]), the majority of plant studies have relied on sequence-matching or tree-based criteria, which do not provide a “no-identification” result when an appropriate match is not present [25]. Therefore, the extent to which the incorporation of a threshold distance affects accuracy of DNA barcoding methods in grasses remains incompletely understood.

DNA barcode datasets that document intraspecific variation hold “untapped” potential for ecological studies that rely on knowledge of genetic diversity of multiple species at community scales [26]. However, wide variation in pairwise intraspecific distances is potentially challenging in a DNA barcoding context [27] as it narrows the barcode gap and potentially the success rate of specimen identifications [5]. Few plant DNA barcoding studies have sampled multiple individuals per species, which is necessary to quantify intraspecific variation [13]. As a result, the accuracy of DNA barcoding methods for plant lineages, including grasses, that are expected to have high levels of intraspecific variation remains poorly understood.

Barcoding markers can reveal genetic variation within sampled individuals to support identification of cryptic or new species [28,29] and in doing so can contribute alongside other data types (e.g. morphological, ecological data) to a truly integrated taxonomy [30,31]. Distance- {e.g. Automatic Barcode Gap Discovery method (ABGD) [32]} and tree-based (e.g. Poisson Tree Processes method [33]) species delimitation methods are available that use individual barcoding markers to partition genetic diversity independently of the taxonomic names that have been assigned to them [25,34]. These species delimitation methods have been applied in only a small number of DNA barcoding studies (e.g. [28,29,35]) and, to our knowledge, have not yet been evaluated in grasses.

A thorough investigation of the accuracy of distance and tree-based DNA barcoding methods is essential to identify potential applications of these methods and their limitations as applied to grasses. Australian Poaceae tribe *Poeae*, which are well characterised in regards to morphological diversity and evolutionary relationships, is an excellent lineage for assessment of these methods in grasses. We generated a reference database of DNA barcode sequence markers for this large and economically significant grass lineage, with comprehensive sampling of both native and exotic taxa at a continental scale. The current study had the following aims: 1. To evaluate the efficacy of official and associated plant DNA barcode markers (*rbc*L, *mat*K, and ITS) for specimen identification using distance- and tree-based methods, and 2. To assess congruence of taxa delimited based on genetic data with current taxa recognized based on morphological characters towards an integrated taxonomic approach for delimitation of native Australian tribe *Poeae* species.

## Materials and methods

### Taxonomic sampling

Nineteen tribe *Poeae s*.*l*. genera and 96 tribe *Poeae* species were sampled in this study (Table 1). This includes 93 (93.9%) of the 99 tribe *Poeae* species accepted as occurring in Australia [2], two *Poa* species (*P*. *hamiltonii* Kirk and *P*. *serpentum* Nees) currently treated as synonyms that potentially warrant recognition as distinct species (R. Soreng, pers. comm., J. Birch, pers. comm., respectively), and one species [*Festuca gautieri* (Hack.) K.Richt.] that is considered potentially invasive in Australia. Field collections were conducted in Australia, with voucher specimens lodged at the National Herbarium of Victoria (MEL). This material was supplemented with herbarium specimens provided by AD, BRI, CANB, HO, MEL, NSW, and PERTH [36]. Herbarium specimens were selected for each species on the basis that the morphological variation and geographic range of each species were broadly sampled. All specimens were determined by taxonomic experts to recognized species using keys provided in Wilson [2] and Vickery [37] prior to inclusion in this study.

**Table 1:** Australian tribe *Poeae* diversity for individual and concatenated DNA barcode markers for distance- (dataset A) and tree- (dataset B) based DNA barcode methods included in this study.

Taxon or clade (number of species/number of individuals)	Species (number of individuals per species)	Number of individuals for individual and concatenated markers^a^^,^ ^b^ (*rbc*L/*mat*K/ITS/*rbc*L+*mat*K/*rbc*L+*mat*K+ITS)
**Ingroup Taxa**		
*tribe Poeae* (96/408)		Dataset A: 391/354/383/395/399 Dataset B: 400/365/393/404/406
*Briza s*.*l*. (3/11)	maxima* (3), minor* (4), subaristata* (4)	11/7/11/11/11
*Briza s*.*s*. (2/7)	maxima* (4), minor* (3)	Dataset A: 7/4/7/7/7 Dataset B: NA
*Catapodium* (2/6)	marinum* (3), rigidum* (3)	6/5/5/6/6
*Chascolytrum* (1/4)	subaristatum* (4)	Dataset A: 4/3/4/4/4 Dataset B: NA
*Cynosurus* (2/6)	cristatus* (n = 3), echinatus* (n = 3)	6/5/5/6/6
*Dactylis* (1/3)	glomerata* (n = 3)	3/3/3/3/3
*Dryopoa* (1/5)	*diva (n = 5)*	5/5/4/5/5
*Festuca* (9/28)	arundinacea* (4), *asperula (4)*, benthamiana (4), gautieri* (1), muelleri (3), nigrescens* (1), *plebeia (4)*, pratensis* (3), rubra* (4)	Dataset A: 26/23/25/26/26 Dataset B: 28/25/27/28/28
*Hainardia* (1/3)	cylindrica* (3)	3/3/3/3/3
*Hookerochloa* (2/7)	*eriopoda (3)*, *hookeriana (4)*	5/5/7/6/7
*Lamarckia* (1/3)	aurea* (3)	3/3/3/3/3
*Lolium* (5/20)	loliaceum* (3), multiflorum* (3), perenne* (9), rigidium* (4), temulentum* (1)	Dataset A: 17/17/16/18/19 Dataset B: 18/18/17/19/19
*Parapholis* (2/9)	incurva* (6), strigosa* (3)	9/8/8/9/9
*Poa* (50/260)	affinis (2), amplexicaulis (4), annua* (5), billardierei (5), bulbosa* (6), cheelii (3), clelandii (6), clivicola (5), compressa* (1), cookii (4), costiniana (6), crassicaudex (4), drummondiana (6), ensiformis (6), fawcettiae (6), fax (5), foliosa (8), fordeana (5), gunnii (6), halmaturina (1), hamiltonii (1), helmsii (5), hiemata (6), homomalla (4), hookeri (5), hothamensis (10), induta (7), infirma* (4), jugicola (5), labillardierei (15), litorosa (3), lowanensis (5), meionectes (5), mollis (4), morrisii (6), orba (2), orthoclada (6), petrophila (3), phillipsiana (6), physoclina (4), poiformis (11), porphyroclados (6), pratensis* (5), rodwayi (4), sallacustris (4),serpentum (2), sieberiana (15), tenera (7), trivialis* (4), umbricola (2)	Dataset A: 254/240/252/256/257 Dataset B: 257/244/255/259/260
*Psilurus* (1/3)	incurvus* (3)	3/3/3/3/3
*Puccinellia* (7/18)	ciliata* (3), distans* (2), fasciculata* (3), longior (1), perlaxa (4), stricta (4), vassica (1)	Dataset A: 16/13/15/16/16 Dataset B: 18/16/17/18/18
*Saxipoa* (1/3)	*saxicola (3)*	3/3/3/3/3
*Sclerochloa* (1/5)	dura* (5)	5/2/5/5/5
*Sphenopus* (1/3)	divaricatus* (3)	3/2/3/3/3
*Sylvipoa* (1/2)	*queenslandica (2)*	2/2/2/2/2
*Vulpia* (5/12)	bromoides* (3), ciliata * (1), fasciculata* (2), muralis* (1), myuros* (5)	Dataset A: 11/5/10/11/12 Dataset B: 12/6/12/12/12
*Fine-leaved clade* (12/33)	Festuca asperula (4), F. benthamiana (4), F. gautieri* (1), F. nigrescens* (1), F. plebeia (4), F. rubra* (4), Psilurus incurvus* (3), Vulpia bromoides* (3), V. ciliata* (1), V. fasciculata* (2), V. muralis* (1), V. myuros* (5)	Dataset A: 28/22/26/28/29 Dataset B: NA
*Broad-leaved clade* (8/30)	Lolium loliaceum* (3), L. multiflorum* (3), L. perenne* (9), rigidium* (4), L. temulentum* (1), Festuca arundinacea* (4), F. muelleri (3), F. pratensis* (3)	Dataset A: 27/26/25/28/28 Dataset B: NA
**Outgroup Taxa**		
*Aegilops*	*comosa*	
*Eremopyrum*	*triticeum*	
*Secale*	*strictum*	

NA, Not applicable.

* Species is exotic in Australia.

^a^ The number of individuals in datasets A and B for each marker differed for some genera as singletons (taxa that were represented by single individuals) were removed prior to distance-based analyses (as outlined in the text).

^b^ Alternative generic circumscriptions for *Briza s*.*s*., *Chascolytrum*, and of *Festuca*, *Lolium*, *Psilurus*, and *Vulpia* into fine-, and broad-leaved clades were included for distance-based analyses (as outlined in the text). Specimen numbers for these genera and clades are provided for dataset A only and are NA for dataset B.

To investigate the impact of generic circumscription on distance-based DNA barcoding results, alternative circumscriptions were compared for genera that are documented in the systematic literature as non-monophyletic or that were non-monophyletic in our phylogenetic analyses (i.e. *Briza* L., *Festuca* L., *Lolium*, *Psilurus* Trin., and *Vulpia* C.C.Gmel.). To achieve monophyly. *Briza subaristata* Lam. was segregated from *Briza s*.*s*. and recognized as *Chascolytrum subaristatum* (Lam.) Desv. following Bayón [38], *Lolium* and three *Festuca* species (*F*. *arundinacea* Schreb., *F*. *muelleri* Vickery, and *F*. *pratensis* Huds.) were placed in a clade, referred to as the “broad-leaved clade”, following Inda *et al*. [39], and the remaining *Festuca* species (*F*. *asperula* Vickery, *F*. *benthamiana* Vickery, *F*. *gautieri*, *F*. *nigrescens* Lam., *F*. *plebeia* R.Br., *F*. *rubra* L.), *Psilurus*, and *Vulpia* were placed in a clade, referred to as the “fine-leaved clade”, following Inda *et al*. [39].

### DNA extraction, amplification, and sequencing

Total genomic DNA was extracted for all species represented in this study from herbarium specimens or silica-preserved samples using a DNeasy Plant Mini Kit (Qiagen, Valencia, California, USA) at Royal Botanic Gardens Victoria or a NucleoSpin® 96 Plant II Core Kit (Machrey-Nagel, Düren, Germany) at Australian Genome Research Facility (AGRF), Adelaide, according to the manufacturer’s protocols. Sequence data for official and associated DNA markers (*rbc*L, *mat*K, and ITS) markers were generated for all individuals. Polymerase chain reaction (PCR) amplification of the chloroplast (*rbc*L and *mat*K) and the nuclear ribosomal (ITS) regions was conducted using a combination of standard and Poaceae-specific primers and methods as outlined in Birch *et al*. [40]. The majority of PCR products were generated at the Royal Botanic Gardens Victoria and were purified and directly sequenced at Macrogen (Seoul, Korea). Samples extracted at AGRF, Adelaide were subsequently sent to AGRF, Brisbane for generation of PCR products and sequencing. Bidirectional sequence chromatograms were edited to produce contiguous sequences in Geneious version 7.0.1 (Biomatters Ltd, Auckland, New Zealand). An alignment was generated using the Geneious alignment function (70% similarity cost matrix with default gap opening settings), which was manually adjusted to improve the alignment for difficult-to-align regions. Where sequence data were not available the sequence was coded as missing (N). Standard ambiguous base calls [41] were applied to polymorphic base pair positions. Sequences, collection data, and voucher specimen images were submitted to Barcode of Life Data Systems (BOLD) to comply with barcode requirements (BOLD accession numbers and voucher specimen collection data are provided in S1 Table).

### Specimen identification: Distance-based

Distance-based barcoding analyses were conducted for the entire tribe *Poeae*, for individual genera, and for alternative circumscriptions of *Briza*, *Chascolytrum*, *Festuca*, *Lolium*, *Psilurus*, and *Vulpia*, as previously outlined. Taxa that were represented by single individuals were removed prior to distance-based barcoding analyses, as without conspecific individuals for comparison, positive identifications were not possible [24]. Pairwise distances were estimated for individual (*rbc*L, *mat*K, ITS) and concatenated {*rbc*L+*mat*K [chloroplast dataset (CH)], *rbc*L+*mat*K+ITS [combined dataset (CO)]} markers using the K80 evolutionary model in Ape version 3.2 [42] in R [43]. For each genus with more than a single species represented in this study, inter- and intra-specific distances for each DNA marker were calculated in Species Identifier v.1.8 [24] and were plotted in ggplot2 [44].

The “nearest neighbour” (NN), “best close match” (BCM), and “threshold ID” (TID) [24] distance-based methods were tested for barcoding efficacy of genera and species using Spider version 1.3–0 [45]. Genetic distance threshold values were tested from 0.001˗2.5% distance in 0.05% intervals for genera and from 0.0001˗2.5% distance in 0.005% intervals for species to identify an optimal threshold that minimized the cumulative error (number of false negatives plus number of false positives). Where no single threshold was optimal (i.e. multiple distances shared the minimum cumulative error), the largest value in the range was selected and applied. Specimen identifications to generic or species rank were considered: 1. “true” in NN when the closest individual to the query was congeneric or conspecific, respectively and “correct” in BCM and TID analyses when all individuals with the closest distance to the query were congeneric or conspecific, respectively, and within the threshold applied; 2. “ambiguous” in BCM analyses when different allogeneric or allospecific individuals, respectively, shared the closest distance to the query and were within the threshold value or in TID analyses when different allogeneric or allospecific individuals, respectively, were within the threshold value; 3. “no identification” in BCM and TID analyses when individuals were genetically more distant to the query than the threshold value; and 4. “false” in NN when the closest individual to the query was allogeneric or allospecific, respectively and “incorrect” in BCM analyses when allogeneric or allospecific individuals, respectively, shared the closest distance to the query and were within the threshold value or in TID analyses when all individuals within the threshold value were allogeneric or allospecific, respectively.

### Specimen identification: Tree-based

Congruence among markers was not assessed prior to their concatenation as this study sought to investigate the placement of individuals for identification, rather than determination of relationships. Molecular phylogenies were reconstructed using the maximum likelihood (ML) criterion in RAxML version 8 [46] and Bayesian inference (BA) in MrBayes 3.2 [47] as outlined in Birch *et al*. [40]. Briefly, the best-fit models of molecular evolution were determined for all datasets using the corrected Akaike information criterion implemented in jModelTest [48,49]. Maximum likelihood reconstructions were conducted using the GTR + Ґ model of evolution, 1000 bootstrap iterations, using the rapid bootstrap analysis and search for the best-scoring tree over a single run. Bayesian inference reconstructions were conducted on individual and concatenated datasets, with the latter dataset partitioned and parameters estimated for each partition. Bayesian analyses were performed using Markov chain Monte Carlo (MCMC) sampling, two independent replicates with a heating temperature of 0.2. Analyses of single- and multi-locus datasets were run for four and eight million generations, respectively, with sampling every 1000 generations, and the initial 25% of trees were considered as burn-in and were discarded. Remaining trees were combined to construct 50% majority-rule consensus trees that were visualized in Figtree v1.3.1 (http://tree.bio.ed.ac.uk/software/figtree/).

For specimen identification based on phylogenetic reconstructions, we applied criteria according to the “liberal” tree-based method of Meier *et al*. [24]. Specimen identifications to generic or species rank were considered: 1. a “success” when the individual was at least one node into a clade exclusively consisting of conspecific individuals, sister to a clade with conspecific individuals, or in a polytomy with conspecific individuals; 2. “ambiguous” if the individual was placed in a polytomy with at least one conspecific and one allospecific individual, sister to a clade with allospecific and conspecific individuals, or if no conspecific individuals were included in the dataset; and 3. “misidentified” if the individual was at least one node into an allospecific clade, sister to a clade with allospecific individuals only, or placed in a polytomy with only allospecific individuals.

### Species discovery: Distance-based

We used ITS data to assess congruence among distance and tree-based species discovery methods as this was the only marker in this study that contained sufficient genetic variation among tribe *Poeae* members to be potentially informative for discovery of genetic entities. Species discovery analyses focused on the native Australian genera within tribe *Poeae* for which comprehensive regional sampling was achieved (i.e. *Festuca*, *Hookerochloa* E.B.Alexeev, *Poa*, and *Puccinellia*). Genetic sequence data for 66 species, recognized based on classical taxonomy of morphological characters and identified using keys provided in Wilson [2] and Vickery [37], were then used as the basis for assessment of congruence with entities recognized based on partitioning of genetic data.

Data for each genus were imported into the online ABGD program interface at http://wwwabi.snv.jussieu.fr/public/abgd/abgdweb.html. Prior intra-specific minimum and maximum diverge values from 0.001 to 0.100, respectively, were applied. These values incorporated the optimal threshold value for the ITS marker for native tribe *Poeae* genera as previously estimated in the Spider package for application in BCM and TID analyses. The K80 evolutionary model was applied as it achieved a better fit to the data, as calculated using the corrected AIC criterion in jModelTest, than the other models available in the software. Transition/transversion ratios in the ITS data of 2.36, 1.6, 1.37, and 3.04 were quantified in jModelTest for *Hookerochloa*, *Festuca*, *Poa*, and *Puccinellia*, respectively, for application in ABGD analyses. Barcode relative gap width values of 0.75, 1.0, and 1.5 were applied to assess the influence of this parameter on the number of entities recognized, with the number of iterations (20) and number of bins (20) held stable.

### Species discovery: Tree-based

The Poisson Tree Processes (PTP) method models speciation using the number of substitutions inferred from branch lengths on a ML input tree [33]. Two independent Poisson processes are estimated representing the distribution of substitutions within and among species branching events [33]. The PTP software [33] version 0.51 was run via the command line. The ML phylogeny generated from the ITS dataset was pruned to remove non-focal genera and a distinct phylogeny was generated for each of the four genera (*Hookerochloa*, *Festuca*, *Poa*, and *Puccinellia*). The Bayesian implementation of the PTP model was run with 1,000,000 iterations, sampling every 1000 generations, discarding the initial 25% of the trees generated that were considered to represent the burn-in stage. Convergence of the MCMC chain was assessed through a visual check of the likelihood plot to ensure stationarity of likelihood values.

## Results

We achieved representation of all tribe *Poeae* species present in Australia with the exception of four *Puccinellia* species that are known only from single or type specimens and two species for which leaf material could not be obtained (*Puccinellia macquariensis* (Cheeseman) Allan & Jansen and *Poa kerguelensis*) (Hook.f.) Steud (Table 1). We included 3–15 individuals per species for 79 species (82.3%) with only 9 (9.4%) and 8 (8.3%) species represented by one or two individuals, respectively. Nineteen genera and 96 ingroup species were represented by 408 individuals in this study (Table 1). The mean number of individuals per *Poa* species was 4.84, and for remaining genera was 3.46 individuals per species. Summary statistics for single- and multi-locus datasets are provided in S2 Table.

### Specimen identification: Distance-based

**Fig 1:**
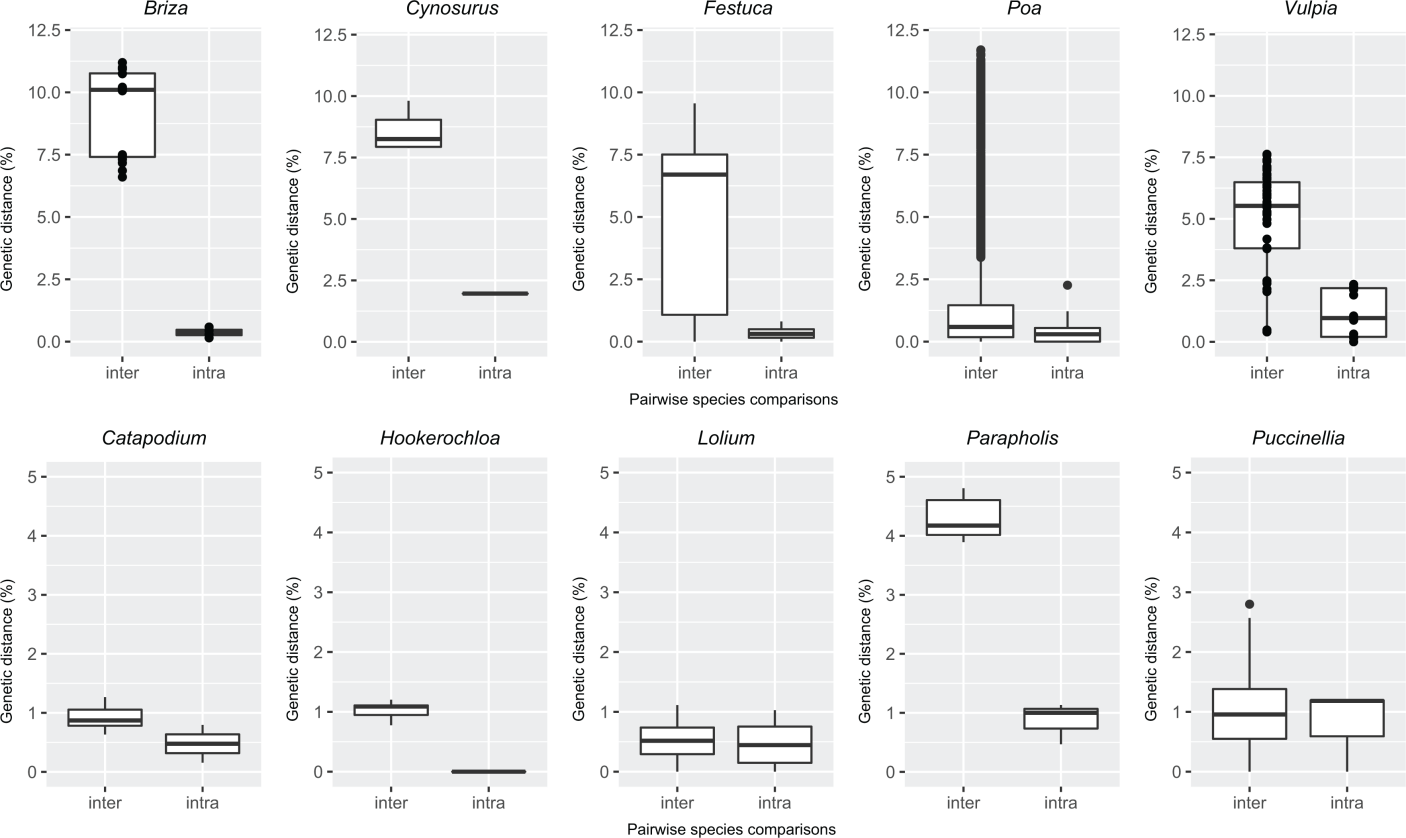
Intra- and inter-specific pairwise genetic distances (K80 model) for the Internal Transcribed Spacer (ITS) marker. Data for all Australian tribe *Poeae* genera with more than two species represented in this study are shown. Inter, Inter-specific; Intra, Intra-specific.

Alignments contained 391, 354, 383, 395, and 399 individuals for *rbc*L, *mat*K, ITS, the CH, and CO datasets, respectively, following removal of taxa represented by single individuals (Table 1 Dataset A, S2 and S3 Tables). Of the markers tested, *rbc*L was the least variable, followed by *mat*K, with ITS the most variable. For *rbc*L, *mat*K, and ITS, mean inter- and intra-specific distances were 0.89 and 0.19, 2.28 and 0.39, and 5.94 and 0.68, respectively. The mean value for inter-specific distances ranged from 0.02% (*Puccinellia*) to 1.03% (*Festuca*) for *rbc*L, from 0.12% (*Lolium*) to 2.54% (*Cynosurus* L.) for *mat*K, and from 0.43% (*Lolium*) to 10.63% (*Briza s*.*s*.) for ITS. Mean inter- and intra-specific distances and ranges of genetic distances varied among tribe *Poeae* genera (Fig 1).

**Fig 2:**
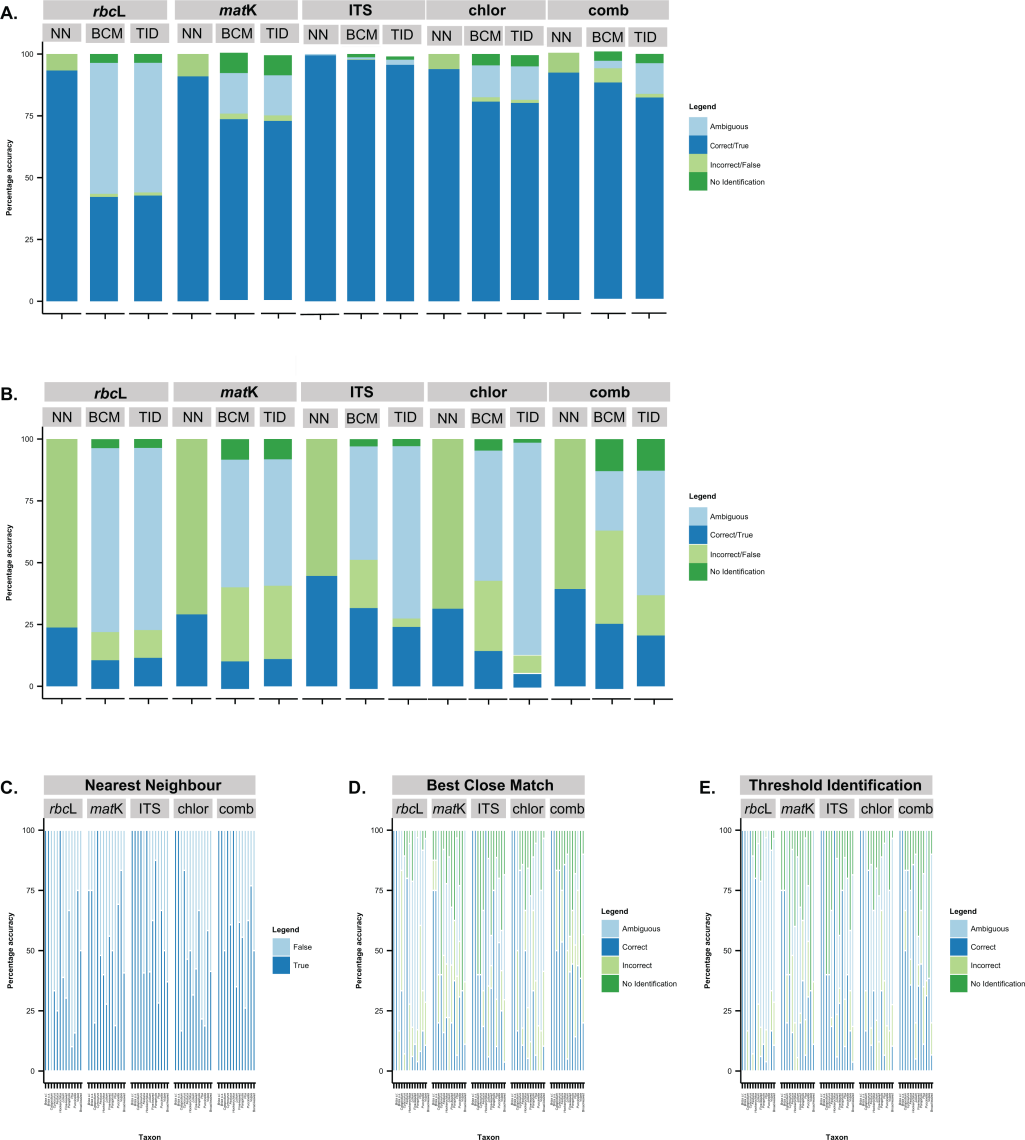
Success rates (percentages) for specimen identification of tribe *Poeae* taxa using distance-based methods. Success rates were calculated for all genera (A.) and species (B.) collectively and for species within single genus datasets (C.–E.) using nearest neighbour (A., B., C.), best close match (A., B., D.), and threshold ID (A., B., E.) methods based on individual (*rbc*L, *mat*K, ITS) and concatenated (*rbc*L+*mat*K, *rbc*L+*mat*K+ITS) DNA barcode markers. BCM, Best close match; Chlor, Chloroplast dataset (*rbc*L+*mat*K); Comb, Combined dataset (*rbc*L+*mat*K+ITS); ITS, Internal transcribed spacer; NN, Nearest neighbour; TID, Threshold Identification.

The NN correctly (i.e. “true” category) identified the largest number of tribe *Poeae* individuals to genus and species for all markers (Fig 2A and 2B, S3 Table). With this method, 91.0–99.5% of individuals were matched to a congener using single- and multi-locus datasets. In comparison, with the estimated optimal threshold values applied, the BCM and TID methods correctly (i.e. “correct” category) identified only 43.5–97.6% and 43.5–96.6% of individuals, respectively, to congeners. When all tribe *Poeae* individuals were included, the NN method correctly (i.e. “true” category) matched 23.8–44.6% of individuals to species rank compared to 11.0–32.4% and 4.2–24.0% of individuals when the BCM and TID methods (i.e. “correct” category), respectively, were applied based on single- and multi-locus datasets.

For all distance-based methods, the most accurate placement of individuals to genera and species, in terms of the number of “true” or “correct” matches, was achieved based on the ITS marker, which outperformed the other individual markers, as well as the CH and CO datasets (Fig 2A–2E, S3 Table). Based on the ITS dataset, 99.5% of individuals were accurately matched to congeners using the NN, compared to 97.6% and 96.6% applying an optimal threshold value of 0.16% within the BCM and TID methods, respectively (Fig 2A, S3 Table). Based on ITS data, when all tribe *Poeae* individuals were included, 44.6%, 32.4%, and 24.0% of individuals were correctly matched to conspecific taxa (i.e. “true” or “correct” categories) when the NN, BCM, and TID methods, respectively, were applied (Fig 2B, S3 Table).

A greater proportion of individuals were correctly matched to species, for BCM and TID analyses when the reference dataset included, and optimal thresholds were calculated for, a single genus or clade (Fig 2C–2E, S3 Table). Based on the ITS dataset and applying the NN method, 100% of *Briza*, *Catapodium* Link, *Cynosurus*, and *Hookerochloa* individuals were accurately matched to species (Fig 2C, S3 Table). For other genera, none of the markers contained sufficient variation to accurately match a large proportion of individuals to species using any of the matching methods (Fig 2C–2E, S3 Table). Based on the ITS dataset and applying the NN method, 71.4% of individuals within the fine-leaved clade; 28.6% of *Poa* individuals; 58.8% of *Puccinellia* individuals; and 38.5% of individuals within the broad-leaved clade were accurately matched to species (Fig 2C, S3 Table).

For both the BCM and TID methods, different optimal threshold values were estimated for specimen identifications at generic and species ranks based on single- and multi-locus datasets for tribe *Poeae* and for individual genera. Based on our reference library, no single genetic distance threshold was identified for individual or concatenated markers for application within TID analyses that differentiated inter- and intra-specific genetic distances, which could serve as a “barcode-gap” for all tribe *Poeae* taxa. A barcode-gap was identified that differentiated *Briza*, *Catapodium*, *Cynosurus*, and *Hookerochloa* species (S3 Table).

### Specimen identification: Tree-based

**Fig 3:**
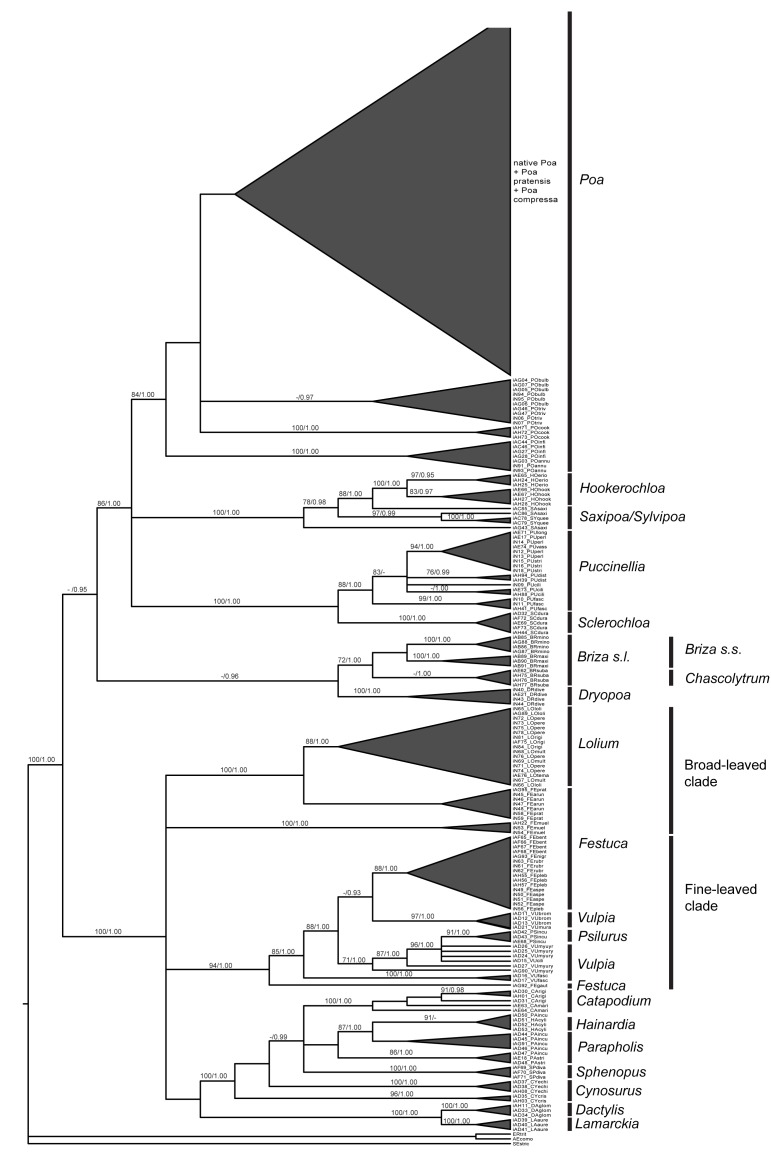
The Bayesian inference of phylogenetic relationships among Australian tribe *Poeae* based on the ITS marker. Support values are provided above the branches including bootstrap (maximum likelihood) and posterior probabilities (Bayesian inference) before and after the forward slash, respectively.

The resulting *rbc*L, *mat*K, ITS, CH and CO datasets included 400, 365, 393, 404 and 406 individuals, respectively (Table 1 Dataset B, S2 and S4 Tables). Of the datasets tested, the Bayesian inference phylogeny based on the ITS (Fig 3) and the combined (S1 Fig) datasets contained the largest number of well-resolved clades with strong support. Only these datasets contained the resolution necessary for application to testing accuracy of placement of individuals using tree-based methods.

In the phylogeny reconstructed from ML and BA searches of the ITS dataset, specimen identification was correct to genus for 97.4% and 96.4% of individuals, respectively, and to species for 28.5% and 26.2%, respectively, recognizing only those clades with greater than 65% bootstrap and 0.95 posterior probabilities (Table 2, S4 Table). In the phylogeny reconstructed from ML and BA searches of the combined dataset, specimen identification was correct to genus for 97.1% and 89.4% of individuals, respectively, and to species for 31.3% and 25.6%, respectively (Table 2). All genera were monophyletic in the ITS and CO phylogenies, with the exception of *Catapodium* (CO phylogeny), *Cynosurus*, *Festuca*, *Hainardia* Greuter, *Parapholis* C.E.Hubb, *Puccinellia* (ML analyses only), *Saxipoa* Soreng, L.J.Gillespie & S.W.L.Jacobs, and *Vulpia* (Fig 3, S1 Fig). In all phylogenies, the fine-leaved *Festuca* were placed with *Vulpia* [94 bootstrap support (BS)/1.00 posterior-probability support (PP)] (Fig 3) and broad-leaved *Festuca* (excluding *Festuca muelleri*) were placed with *Lolium* (100 BS/1.00 PP) (Fig 3).

**Table 2:** Success rates (percentages) for specimen identification based on individual (ITS) and concatenated (*rbc*L+*mat*K+ITS) DNA barcode markers using distance-based (nearest neighbour and best-close match) and tree-based (maximum likelihood and Bayesian inference) methods.

Taxon	Number of taxa	Specimen identification (%)		Specimen identification (%		Specimen identification (%)		Specimen identification (%)	
	(morphology)	(ITS, N = 18 genera, 81 species)^a^		(*rbc*L+*mat*K+ITS, N = 18 genera, 82 species)		(ITS, N = 19 genera, 96 species)		(*rbc*L+*mat*K+ITS, N = 19 genera, 96 species)	
		NN	BCM	NN	BCM	ML	BA	ML	BA
		(true/false)	(correct/incorrect/ambiguous/no identification)	(true/false)	(correct/incorrect/ambiguous/no identification)	correct/incorrect/ambiguous	correct/incorrect/ambiguous	correct/incorrect/ambiguous	correct/incorrect/ambiguous
Genera	19	**99.5**/0.5^b^	**97.6**/0.3/0.8/1.3	**92.0**/8.0	**87.5**/5.8/3.0/3.7	**97.4**/0.8/1.8^c^	**96.4**/0.5/3.1	**97.1**/1.2/1.7	**89.4**/0.7/9.9
Species	96	**44.6**/55.4	**32.4**/18.5/46.2/2.9	**39.3**/60.7	**26.1**/37.3/23.8/12.8	**28.5**/6.4/65.1	**26.2**/4.1/69.7	**31.3**/4.9/63.8	**25.6**/4.9/69.5

BA, Bayesian inference; BCM, Best close match; ITS, Internal transcribed spacer; ML, Maximum likelihood; N = Sample size; NN, Nearest neighbour.

^a^ The number of genera and species for each marker differed for distance- and tree-based analyses as alternative generic circumscriptions for *Briza s*.*s*., *Chascolytrum*, and of *Festuca*, *Lolium*, *Psilurus*, and *Vulpia* into fine-, and broad-leaved clades were applied and singletons (taxa that were represented by single individuals) were removed for distance-based analyses (as outlined in the text).

^b^ Percentages of “true” and “correct” specimen identifications are indicated in bold.

^c^ In phylogenetic analyses, clades receiving 65% bootstrap support in maximum likelihood searches or 0.95 posterior probabilities in Bayesian inference searches were recognised.

### Species discovery: Distance- and tree-based

**Fig 4:**
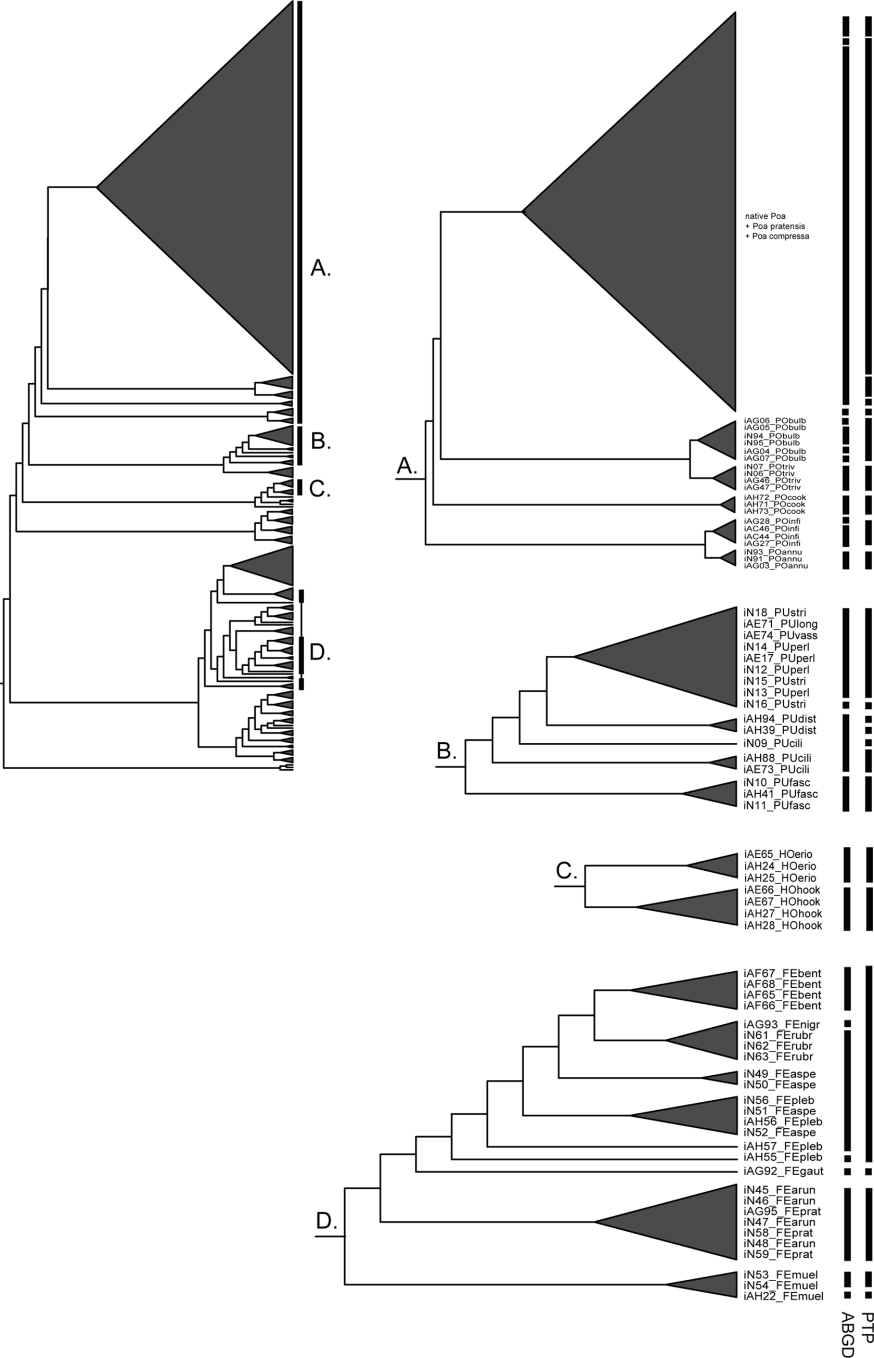
Molecular entities distinguished based on genetic sequence data from the ITS marker estimated by ABGD and PTP methods. Analyses were completed for Australian tribe *Poeae* genera with more than two native species. The maximum likelihood phylogeny for Australian tribe *Poeae* based on the ITS dataset is inset in the top left corner with genera analysed indicated with uppercase letters and shown in detail. Genetic entities are indicated by a black bar to the right of the individuals contained within that entity for ABGD (left) and PTP (right) analyses. ABGD, Automated barcode gap discovery; ITS, Internal transcribed spacer; PTP, Poisson tree processes.

The ABGD method distinguished 8, 2, 13, and 4 entities for *Festuca*, *Hookerochloa*, *Poa*, and *Puccinellia*, respectively (Fig 4, Table 3). Congruent results were obtained for the three gap width values that were investigated (0.75, 1.0, 1.5) for *Hookerochloa* (2 entities) and *Puccinellia* (4 entities). For *Festuca* and *Poa*, 5 and 9 entities, respectively, were distinguished when a gap value of 1.5 was applied, which was slightly fewer than the 8 and 13 entities, respectively, that were distinguished when gap values of 1.0 and 1.5 were applied. For all genera, the results for the intraspecific divergence value that was closest to the optimal threshold value calculated for distance-based specimen identification methods were selected. The PTP model distinguished 5, 2, 10, and 7 entities for *Festuca*, *Hookerochloa*, *Poa*, and *Puccinellia*, respectively (Fig 4, Table 3). Support values (posterior probabilities for the entities as distinct clusters) ranged from 0.0 to 1.00. The ABGD method and the PTP model delimited 9 genetic entities that were congruent with recognized species (13.6%), individuals of 49 species (74.2%) were placed in a genetic entity with allospecific individuals, individuals of three (4.6%) species were split into distinct genetic entities, and individuals from 5 (7.6%) species had some individuals placed into distinct entities and others combined into a genetic entity with allospecific individuals.

**Table 3:** Species delimitations for Australian tribe *Poeae* genera containing greater than two native species using morphological and genetic sequence data from the ITS marker estimated by ABGD and PTP methods.

Taxon	Number of species	Number of individuals	ABGD^a^^b^	PTP^a^^b^
***Festuca***	**9**		**N(8)**^**c**^	**N(5)**^**c**^
F. arundinaceae*		4	L^1^	L^1^
*F*. *asperula*		3	L^2^	L^2^
*F*. *benthamiana*		4	+	L^2^
F. gautieri*		1	+	+
*F*. *muelleri*		3	S^3,4^	S^3,4^
F. nigrescens*		1	+	L^2^
*F*. *plebeia*		4	L/S^2,5^	L^2^
F. pratensis*		3	L^1^	L^1^
F. rubra*		3	L^2^	L^2^
***Hookerochloa***	**2**		**N(2)**	**N(2)**
*H*. *eriopoda*		3	+	+
*H*. *hookeriana*		4	+	+
***Poa***	**50**		**N(13)**	**N(10)**
**Natives**				
*P*. *cookii*		4	L/S^6,7^	L/S^5,9^
*P*. *foliosa*		8	L/S^7,8^	L/S^6,9^
*Poa* native clade		216	L/S^7,9^	L/S^7,8,9^
**Exotics**				
P. annua*		4	+	+
P. bulbosa*		6	S^12, 13,14^	+
P. compressa*		1	+	+
P. infirma*		4	S^10, 11^	+
P. pratensis*		5	L^7^	L/S^8,9^
P. trivialis*		4	+	+
***Puccinellia***	**7**		**N(4)**	**N(7)**
P. ciliata*		3	L^15^	S^10,11^
P. distans*		2	L^15^	S^12,13^
P. fasciculata*		3	+	+
*P*. *longior*		1	L^16^	L^14^
*P*. *perlaxa*		4	L^16^	L^14^
*P*. *stricta*		3	L/S^16,17^	L/S^14,15^
*P*. *vassica*		1	L^16^	L^14^

ABGD, Automated barcode gap discovery; ITS, Internal transcribed spacer; PTP, Poisson tree processes.

* Species is exotic in Australia.

^a^ L refers to a genetic entity that included more than one species; S refers to a single species that was split into more than one genetic entity; + refers to a genetic entity that was congruent with the species.

^b^ Within a column, species that share a superscript letter were delimited as a single genetic entity.

^c^ N (#) refers to the number of genetic entities delimited.

## Discussion

### Taxonomic considerations

Poaceae tribe *Poeae* is a particularly challenging lineage for species identification and delineation [50,51], and as such provides a rigorous test of DNA barcoding methods. DNA barcoding for identification purposes relies heavily on taxonomy. Taxonomic concepts can be malleable and establishing a clear taxonomic hierarchy for the group under study is not always straightforward. We achieved comprehensive sampling of tribe *Poeae* species present in Australia and included multiple individuals per taxon to ensure intraspecific variation for the majority of tribe *Poeae* species was captured.

Achieving accurate identifications based on genetic data for taxa that do not resolve as monophyletic is difficult using DNA barcoding methods as individuals may be genetically more similar to those of a different species [52]. A greater number of specimen identifications were “correct” to species rank when *Festuca* and *Vulpia* species were treated as members of fine- or broad-leaved clades. The fine- and broad-leaved clades had narrower pairwise genetic distances ranges than those of both genera. This result is likely to reflect the greater accuracy of barcode gap or the threshold genetic distance estimates for entities with narrow and non-overlapping ranges of inter- and intraspecific variation. Meyer and Paulay [27] also noted this for cowries; when taxonomy did not reflect evolutionary significant units, error rates of identification success increased due to increases in the ranges of intraspecific variation and interspecific divergence.

We investigated the taxonomic signal present in sequence data from DNA barcode markers to assess congruence with species hypotheses based on morphology. The species discovery methods applied achieved broadly congruent results in the genetic entities that were differentiated based on ITS data. Two genetic entities were distinguished for *Hookerochloa* that were congruent with recognized species. For the remaining genera, the numbers of entities distinguished based on genetic data were fewer than the number of recognized species. For native tribe *Poeae* species, the species discovery methods applied did not reveal the presence of cryptic genetic variation. Overwhelmingly the genetic data merged species that are otherwise recognized as distinct based on morphological and ecological characters. These results indicate that for tribe *Poeae*, and likely for other grass lineages, sequence data in the standard DNA barcode markers are not variable enough for species discovery, particularly given the extensive infraspecific variation and shared polymorphisms among species as documented in this study. A study currently underway that has generated genomic data obtained using a genome skimming (RADseq) approach for Australian *Poa* suggests that this approach captures sufficient genetic variation to assess species boundaries.

An integrated taxonomic approach applies multiple lines of evidence to understand the origin and evolution of species [53]. For Australian tribe *Poeae* both morphological [37,50] and genetic data (this study) reveal extensive infraspecific variation. For tribe *Poeae* the genetic entities recognized by species discovery methods were broadly congruent with clades recovered in the ML phylogeny based on ITS data including for polyphyletic species. This observed congruence suggests that the species discovery methods may hold potential for preliminary, rapid assessment of distinct genetic groups and detection of genetically distant individuals, which can then be assessed in conjunction with morphological data. Greater confidence in taxonomic boundaries results where congruence of datasets is documented and where any discordance can be explained within the context of evolutionary history [53].

### DNA barcode markers

Across all methods investigated, greatest resolution of genera and species was achieved based on the ITS marker. Other studies (for example, [12,15]) have also indicated that ITS is an informative marker for DNA barcoding of grasses. However, the presence of multiple paralogous ITS copies [54], may complicate the interpretation of genetic distances among and within species. For this reason we consider its use, alongside other markers, representing chloroplast and nuclear genomes, to be more optimal than its use individually. Additional resolution may be achieved by inclusion of additional highly variable markers into the reference database (for example, *rpl*32-*trn*L as per [12]). It is clear that the low levels of genetic diversity in both *rbc*L and *mat*K render these markers uninformative as DNA barcodes for specimen identification of Australian tribe *Poeae* species. The multi-locus chloroplast dataset (*rbc*L+*mat*K) provided only marginally greater resolution of taxa over that achieved based on the individual *mat*K region, consistent with findings in other plant studies [7,10,21].

We tested for the presence of a single genetic distance threshold that served to distinguish tribe *Poeae* genera and species. Even within this tribe, the optimal genetic distance threshold values estimated from our reference database varied among genera and it was not possible to identify a single genetic distance threshold for integration into distance-based specimen identification methods. Our results suggest that threshold values will need to be calculated on a taxon–by-taxon and marker-by-marker basis, rather than being universally applied to distinguish species across multiple genera and lineages.

A genus-specific barcode gap was identified for genera represented in the Australian flora by a small number of species (for example, *Briza*, *Catapodium*, *Cynosurus*, and *Hookerochloa*). The first three of these genera are exotic in Australia; therefore, while their sampling is complete for Australia it is incomplete in terms of their global diversity. As a result, the inter-specific distances among species in these genera may be larger due to the absence of sister taxa, creating an “artificial” or regional barcode gap [27]. Conversely, for large genera such as *Poa*, that contain many closely related species in the Australian flora, even applying the most variable marker (ITS), no genetic distance threshold was identified for accurate identification of specimens to species rank. DNA barcoding studies, such as this one, that achieve comprehensive sampling of closely related species typically show the lowest levels of species resolution [21]. Our study suggests that the official DNA barcode markers (or markers with equivalent levels of genetic variation) do not provide a reliable tool for accurate identification of specimens to species rank or for quantification of species numbers within floras that include grass genera represented by moderate to high species numbers.

A significant overlap was observed between intra- and interspecific genetic distances for all markers studied due to the presence of a large number of outlier pairwise intraspecific genetic distances. For the distance-based methods investigated, identification is based on the minimum rather than the average pairwise genetic distance (i.e. assignment of identity based on the *nearest* to the query individual rather than on the calculation of *mean* values for conspecific or heterospecific individuals). Therefore, accurate identifications remain possible despite overlap between intraspecific and interspecific genetic distances. Simulation studies have indicated that while the mean rate of identification success decreases as overlap of distance distributions of a query sequence to conspecific and heterospecific sequences increases, this parameter alone remains a poor predictor of identification success [52].

### Specimen identification: distance- versus tree-based methods

The percentage of accurate identification for tribe *Poeae* genera based on the ITS marker when distance-based methods were applied (96.6–99.5%) were consistent with percentage of generic resolution in other studies (for example, [13,21]). The ability to provide an accurate generic determination for an unknown individual is particularly valuable for grasses where fragmentary samples (e.g. herbivore stomach contents, environmental samples, or leaf samples) are common. These would not otherwise be able to be identified as the morphological characters required for determination are either not visible or are absent. Additionally, identification of plant material from horticultural sources or in living collections for which provenance is unknown, can be very challenging as identification keys spanning global taxonomic coverage are not always available (V. Stajzic, pers. comm.). A combined approach using DNA barcoding methods for generic determination and morphology based taxonomic keys for species determination may be an efficient use of taxonomic resources.

The percentage of identification success for tribe *Poeae* species (24.0%–44.6%) was well below that achieved based on the ITS marker for other lineages, with 100% of species matching based on BLAST searches for Australasian *Austrostipa* (Poaceae) [15], 96% discrimination of *Dinebra* species (subf. Chloridoideae) [12] and 74.2% species differentiation across monocots [13]. The low levels of sequence divergence observed for *Poa* within the markers applied likely reflects its recent radiation in Australia; the most recent common ancestor of all but one species [*P*. *cookii* (Hook.f.) Hook.f.] was estimated to have diversified from only 3.9 (HPD values: 2.1–6.0) million years onwards [40]. Incomplete lineage-sorting and interspecific hybridisation events may also contribute to the failure of DNA barcodes to accurately resolve taxa that have only recently diverged [55]. Additional resolution may be achieved from concatenation of multiple markers [56], however, this may not always be the case. Determination accuracy was lower based on our combined (rbcL+matK+ITS) dataset than that of our ITS datasets (Table 3). Other studies that also have found that the concatenation of loci does not always improve resolution power [55].

For tribe *Poeae* and most individual genera, the NN method correctly matched congeneric and conspecific individuals more frequently than the BCM and TID methods. The NN method has been documented as robust and the most consistently performing method for specimen matching in a DNA barcoding context based on both real [15,23] and simulated [23] data. However, the smallest number of “incorrect” identifications was observed for the TID method over the BCM and NN methods. This reflects the relatively large number of tribe *Poeae* individuals with pairwise genetic distances that were statistical outliers, which were categorized as “false” based on the NN method, but, as they were outside the generic threshold value/s applied, as “no identification” based on the BCM and TID methods. The TID method applies more stringent criteria for identification and produces fewer “incorrect” identifications as a result, with the “cost” of this stringency being the generation of fewer “correct” identifications. The variable stringencies of these different methods provide an opportunity to apply the method that best aligns with the requirements of the barcoding application. For applications requiring minimization of false positive identifications, for example, for biosecurity applications such as screening for invasive species, the TID method may be the most appropriate.

Tree-based methods provided successful identification of the majority of the exotic tribe *Poeae* species present in Australia that were represented by more than one individual in this study. This ability to accurately identify material from many non-native tribe *Poeae* species, increases the potential contribution of this reference dataset for applications that would benefit from potentially rapid identification of these species, including from fragmentary material, such as biosecurity, forensic, and horticultural applications. The application of this reference database for these purposes would be further improved by increased sampling for those species currently represented by single individuals (*Festuca gautieri*, *F*. *nigrescens*, *Poa compressa* L., *and Lolium temulentum* L.) and by incorporation of other global tribe *Poeae* species that present the risk of invasion into Australia.

Our analyses suggest that a similar percentage of specimens were accurately identified to species rank using tree- (25.6–31.3%) and distance-based (26.1–44.6%) methods. However, the smallest percentage of “incorrect” identifications to species rank was achieved by the most stringent distance-based TID method (3.4–28.8%) followed by the tree-based methods (4.1–6.4%). Simulation studies have also suggested that distance- and tree-based barcoding methods achieve similar levels of identification success [23,52], despite the incorporation of more complex models of nucleotide evolution for placement of individuals based on tree-based methods. Additional computation time is typically required for tree- over distance-based analyses, which may not be warranted for lineages in which little additional resolution is achieved by tree- over distance-based methods.

## Conclusions

We have generated a tribe *Poeae* reference library and new sequence data for the official plastid barcoding (*rbc*L and *mat*K) and associated (ITS) markers with comprehensive representation across the Australian continent. Using the ITS dataset, for tribe *Poeae* in Australia we were able to correctly identify 97.6% of individuals to genera and 32.4% of individuals to species, based on the BCM distance-based method. The nearest neighbour method provided a higher percentage of “correct” specimen identifications, but suffered from a larger number of “incorrect” identifications at the species rank. The TID method typically provided the lowest percentage of “incorrect” specimen determinations with the “cost” of the stringency in the criteria applied being a lower percentage of “correct” identifications. A barcode gap that facilitated determination of species was identified for smaller genera of tribe *Poeae*, including *Briza*, *Catapodium*, *Cynosurus*, and *Hookerochloa*. Based on the ITS dataset and applying the liberal tree-based method to assess the maximum likelihood phylogeny we were able to correctly identify 97.4% of individuals to genera and 28.5% of individuals to species. Tree-based methods correctly identified almost all exotic species, including those in genera containing native species i.e. *Poa*, *Festuca*, and *Puccinellia*

These investigations revealed a number of issues that prevent accurate identification of Australian tribe *Poeae* species using current barcoding methods. Different genetic distance threshold values were identified as optimal for different genera and barcoding markers, meaning that these values needed to be estimated independently for each genus or clade. For the markers tested in this study, tribe *Poeae* species in Australia have wide pairwise genetic distances ranges, with intraspecific distances that include a large number of statistical outliers. For these and other groups with wide pairwise genetic distances ranges, only a subset of individuals will be correctly identified to species. Australian tribe *Poeae* lineages represent recent radiations and the genetic variation contained within even the most variable marker investigated here was insufficient for accurate identification of many *Festuca*, *Poa*, *and Puccinellia* species. For recently diverged species the challenges for application of both genetic and morphological data to species identification and delimitation are similar, including lack of sufficient variation for accurate resolution, homoplasy, and, potentially, incongruent taxonomic signal across characters, data partitions, or data types.

## Supporting information

S1 Table Voucher specimen data for individuals, presence or absence of sequence data in individual and concatenated DNA barcode markers, Barcode Of Life Data System (BOLD) reference numbers, and presence or absence of voucher specimen images. AD, State Herbarium of South Australia; BRI, Queensland Herbarium; CANB, Australian National Herbarium; HO, Tasmanian Museum and Art Gallery; ITS, Internal transcribed spacer; MEL, Royal Botanic Gardens Victoria; N, Absent; NSW, Royal Botanic Gardens and Domain Trust; PERTH, Western Australian Herbarium; Y, Present. ^a^Herbarium abbreviations follow Theirs (continuously updated).
(PDF)

S2 Table Summary statistics and sequence quality of individual and concatenated DNA barcode markers for specimen identification and species discovery based on distance- (dataset A) and tree- (dataset B) based methods. (PDF)

S3 Table Success rates (percentages) for specimen identification using distance-based methods (nearest neighbour, best close match, and threshold ID [24], as outlined in the text) based on individual (ITS) and concatenated (*rbc*L+*mat*K, *rbc*L+*mat*K+ITS) DNA barcode markers. BCM, Best close match; ITS, Internal transcribed spacer; NN, Nearest neighbour; TID, Threshold Identification. ^a^Markers for which no single threshold was optimal across the range tested and for which a default value of 0.100% was applied are indicated with an asterisk (*). ^b^Percentages of “true” and “correct” identifications are indicated in bold.
(PDF)

S4 Table Success rates for specimen identification using tree-based (maximum likelihood or Bayesian inference phylogenies with specimens identified according to the “liberal” tree-based method of Meier *et al*. [24], as outlined in the text) methods for individual (ITS) and concatenated (*rbc*L+*mat*K+ITS) DNA barcode markers. BA, Bayesian inference; ITS, Internal transcribed spacer; ML, Maximum likelihood. ^a^Success rates for generic determinations are indicated in bold. * Taxon represented by a single individual (singleton).
(PDF)

S1 Fig The Bayesian inference of phylogenetic relationships among Australian tribe *Poeae* based on the concatenated (*rbcL*+*mat*K+ITS) DNA barcode markers. Support values are provided above the branches including bootstrap (maximum likelihood) and posterior probabilities (Bayesian inference) before and after the forward slash, respectively.
(EPS)
