# Supplementary material for: Testing efficacy of distance and tree-based methods for DNA barcoding of grasses (Poaceae tribe Poeae) in Australia
Source: PLoS One. 2017 Oct 30;12(10):e0186259. doi: 10.1371/journal.pone.0186259 (PMC5662090; doi:10.1371/journal.pone.0186259)
Supplement: S1 Table — AD, State Herbarium of South Australia; BRI, Queensland Herbarium; CANB, Australian National Herbarium; HO, Tasmanian Museum and Art Gallery; ITS, Internal transcribed spacer; MEL, Royal Botanic Gardens Victoria; N, Absent; NSW, Royal Botanic Gardens and Domain Trust; PERTH, Western Australian Herbarium; Y, Present. aHerbarium abbreviations follow Theirs (continuously updated). (PDF) [file pone.0186259.s001.pdf]

S1 Table

Taxon	Individual reference code	Herbarium accession number[a]	BOLD reference number	*rbc*L	*mat*K	ITS	Chloroplast (*rbc*L+*mat*K)	Combined (*rbc*L+*mat*K+ITS)	Image
Briza maxima L.	iAB89_maxi072	MEL 2037072	AUSG1362-10	Y	Y	Y	Y	Y	Y
Briza maxima L.	iAB90_maxi777	MEL 1588777	AUSG1363-10	Y	Y	Y	Y	Y	Y
Briza maxima L.	iAB91_maxi062	MEL 2015062	AUSG1364-10	Y	Y	Y	Y	Y	Y
Briza minor L.	iAB85_mino727	MEL 225727	AUSG1358-10	Y	N	Y	Y	Y	Y
Briza minor L.	iAB86_mino734	MEL 225734	AUSG1359-10	Y	Y	Y	Y	Y	Y
Briza minor L.	iAG87_mino953	MEL 2355953	AUSG2101-14	Y	N	Y	Y	Y	Y
Briza minor L.	iAG88_mino966	MEL 2355966		Y	N	Y	Y	Y	N
Briza subaristata Lam.	iAE62_subu757	MEL 2269757	AUSG2056-14	Y	Y	Y	Y	Y	Y
Briza subaristata Lam.	iAH75_suba867	CANB 525867	AUSG2198-14	Y	N	Y	Y	Y	Y
Briza subaristata Lam.	iAH76_suba677	CANB 637677	AUSG2204-14	Y	Y	Y	Y	Y	Y
Briza subaristata Lam.	iAH77_suba698	CANB 509698	AUSG2196-14	Y	Y	Y	Y	Y	Y
Catapodium marinum (L.) C.E.Hubb.	iAD28_mari911	MEL 647911	AUSG1148-10	Y	N	N	Y	Y	Y
Catapodium marinum (L.) C.E.Hubb.	iAE63_mari057	HO 501057	AUSG2227-14	Y	Y	Y	Y	Y	Y
Catapodium marinum (L.) C.E.Hubb.	iAE64_mari211	HO 442211	AUSG2226-14	Y	Y	Y	Y	Y	Y
Catapodium rigidum (L.) C.E.Hubb.	iAD30_rigi010	MEL 2014010	AUSG1150-10	Y	Y	Y	Y	Y	Y
Catapodium rigidum (L.) C.E.Hubb.	iAD31_rigi640	MEL 2181640	AUSG1151-10	Y	Y	Y	Y	Y	Y
Catapodium rigidum (L.) C.E.Hubb.	iAH01_rigi015	MEL 2029015	AUSG2045-14	Y	Y	Y	Y	Y	Y
Cynosurus cristatus L.	iAD35_cris303	MEL 2050303	AUSG1155-10	Y	Y	Y	Y	Y	Y
Cynosurus cristatus L.	iAD36_cris155	MEL 225155	AUSG1156-10	Y	Y	N	Y	Y	Y
Cynosurus cristatus L.	iAH03_cris035	MEL 2296035	AUSG2059-14	Y	Y	Y	Y	Y	Y
Cynosurus echinatus L.	iAD37_echi744	MEL 2194744	AUSG1157-10	Y	Y	Y	Y	Y	Y
Cynosurus echinatus L.	iAD38_echi105	MEL 1555105	AUSG1158-10	Y	Y	Y	Y	Y	Y
Cynosurus echinatus L.	iAH08_echi468	MEL 2018468	AUSG2038-14	Y	N	Y	Y	Y	Y
Dactylis glomerata L.	iAD33_glom280	MEL 1582280	AUSG1153-10	Y	Y	Y	Y	Y	Y
Dactylis glomerata L.	iAD34_glom156	MEL 2042156	AUSG1154-10	Y	Y	Y	Y	Y	Y
Dactylis glomerata L.	iAH11_glom390	MEL 2059390	AUSG2050-14	Y	Y	Y	Y	Y	Y
Dryopoa dives (F.Muell.) Vickery	iAE21_dive932	MEL 2355932	AUSG2087-14	Y	Y	Y	Y	Y	Y
Dryopoa dives (F.Muell.) Vickery	iN40_dive052	MEL 2213052	AUSG1073-10	Y	Y	Y	Y	N	Y
Dryopoa dives (F.Muell.) Vickery	iN43_dive904	MEL 2280904	AUSG1075-10	Y	Y	Y	Y	Y	Y
Dryopoa dives (F.Muell.) Vickery	iN44_dive412	MEL 2062412	AUSG1076-10	Y	Y	Y	Y	Y	Y
Dryopoa dives subsp. A	iN42_dive576	MEL 596576		Y	Y	N	Y	Y	N
Festuca aff. asperula	iN52_aspe315	MEL 2037315	AUSG1084-10	Y	Y	Y	Y	Y	Y
Festuca arundinacea Schreb.	iN45_arun731	MEL 269731	AUSG1077-10	Y	N	Y	Y	Y	Y
Festuca arundinacea Schreb.	iN46_arun404	MEL 2274404	AUSG1078-10	Y	Y	Y	Y	Y	Y
Festuca arundinacea Schreb.	iN47_arun597	MEL 2105597	AUSG1079-10	Y	Y	Y	Y	Y	Y
Festuca arundinacea Schreb.	iN48_arun054	MEL 2331054	AUSG1080-10	Y	Y	Y	Y	Y	Y
Festuca asperula Vickery	iN49_aspe977	MEL 285977	AUSG1081-10	Y	Y	Y	Y	Y	Y
Festuca asperula Vickery	iN50_aspe236	MEL 2314236	AUSG1082-10	Y	N	Y	Y	Y	Y
Festuca asperula Vickery	iN51_aspe915	MEL 2275915	AUSG1083-10	Y	Y	Y	Y	Y	Y
Festuca benthamiana Vickery	iAF65_bent230	AD 99823230	AUSG2180-14	Y	Y	Y	Y	Y	Y
Festuca benthamiana Vickery	iAF66_bent100	AD 99744100	AUSG2179-14	Y	Y	Y	Y	Y	Y
Festuca benthamiana Vickery	iAF67_bent779	AD 226779	AUSG2172-14	Y	Y	Y	Y	Y	Y

Taxon	Individual reference code	Herbarium accession number[a]	BOLD reference number	*rbc*L	*mat*K	ITS	Chloroplast (*rbc*L+*mat*K)	Combined (*rbc*L+*mat*K+ITS)	Image
Festuca benthamiana Vickery	iAF68_bent336	AD 235336	AUSG2173-14	Y	Y	Y	Y	Y	Y
Festuca gautieri (Hack.) K.Richt.	iAG92_gaut928	MEL 2359928	AUSG2123-14	Y	Y	Y	Y	Y	Y
Festuca muelleri Vickery	iAH22_muel372	MEL 2357372	AUSG2111-14	Y	Y	Y	Y	Y	Y
Festuca muelleri Vickery	iN53_muel075	MEL 2293075	AUSG1085-10	Y	Y	Y	Y	Y	Y
Festuca muelleri Vickery	iN54_muel310	MEL 2314310	AUSG1086-10	Y	Y	Y	Y	Y	Y
Festuca nigrescens Lam.	iAG93_nigr931	MEL 2359931	AUSG2126-14	Y	Y	Y	Y	Y	Y
Festuca plebeia R.Br.	iAH55_pleb591	HO 546591	AUSG2231-14	Y	N	Y	Y	Y	Y
Festuca plebeia R.Br.	iAH56_pleb982	HO 547982	AUSG2232-14	Y	Y	Y	Y	Y	Y
Festuca plebeia R.Br.	iAH57_pleb226	HO 523226	AUSG2228-14	Y	Y	Y	Y	Y	Y
Festuca plebeia R.Br.	iN56_pleb516	MEL 2131516	AUSG1088-10	Y	Y	Y	Y	Y	Y
Festuca pratensis Huds.	iAG95_prat932	MEL 2359932	AUSG2127-14	Y	Y	Y	Y	Y	Y
Festuca pratensis Huds.	iN58_prat993	MEL 2274993	AUSG1090-10	Y	Y	Y	Y	Y	Y
Festuca pratensis Huds.	iN59_prat071	MEL 273071	AUSG1091-10	Y	Y	Y	Y	Y	Y
Festuca rubra L.	iN60_rubr408	MEL 653408	AUSG1092-10	Y	Y	N	Y	Y	Y
Festuca rubra L.	iN61_rubr802	MEL 2100802	AUSG1093-10	Y	Y	Y	Y	Y	Y
Festuca rubra L.	iN62_rubr513	MEL 2323513	AUSG1094-10	Y	Y	Y	Y	Y	Y
Festuca rubra L.	iN63_rubr827	MEL 1605827	AUSG1095-10	Y	Y	Y	Y	Y	Y
Hainardia cylindrica (Willd.) Greuter	iAD51_cyli507	MEL 2330507	AUSG1171-10	Y	Y	Y	Y	Y	Y
Hainardia cylindrica (Willd.) Greuter	iAD52_cyli607	MEL 2097607	AUSG1172-10	Y	Y	Y	Y	Y	Y
Hainardia cylindrica (Willd.) Greuter	iAD53_cyli947	MEL 2134947	AUSG1173-10	Y	Y	Y	Y	Y	Y
Hookerochloa eriopoda (Vickery) S.W.L.Jacobs	iAE65_erio383	MEL 2025383	AUSG2042-14	Y	Y	Y	Y	Y	Y
Hookerochloa eriopoda (Vickery) S.W.L.Jacobs	iAH24_erio205	MEL 2236205	AUSG2054-14	Y	Y	Y	Y	Y	Y
Hookerochloa eriopoda (Vickery) S.W.L.Jacobs	iAH25_erio400	MEL 2253400	AUSG2055-14	Y	Y	Y	Y	Y	Y
Hookerochloa hookeriana (F.Muell. ex Hook.f.) E.B.Alexeev	iAE66_hook238	MEL 2026238	AUSG2044-14	N	Y	Y	Y	Y	Y
Hookerochloa hookeriana (F.Muell. ex Hook.f.) E.B.Alexeev	iAE67_hook454	MEL 1612454		N	N	Y	N	Y	N
Hookerochloa hookeriana (F.Muell. ex Hook.f.) E.B.Alexeev	iAH27_hook511	MEL 2031511	AUSG2046-14	Y	N	Y	Y	Y	Y
Hookerochloa hookeriana (F.Muell. ex Hook.f.) E.B.Alexeev	iAH28_hook767	MEL 2057767	AUSG2049-14	Y	Y	Y	Y	Y	Y
Lamarckia aurea (L.) Moench	iAD39_aure308	MEL 2293308	AUSG1159-10	Y	Y	Y	Y	Y	Y
Lamarckia aurea (L.) Moench	iAD40_aure109	MEL 2136109	AUSG1160-10	Y	Y	Y	Y	Y	Y
Lamarckia aurea (L.) Moench	iAD41_aure294	MEL 2339294	AUSG1161-10	Y	Y	Y	Y	Y	Y
Lolium loliaceum (Bory & Chaub. ex Fauché) Hand.-Mazz.	iAG89_loli952	MEL 2355952	AUSG2100-14	Y	Y	Y	Y	Y	Y
Lolium loliaceum (Bory & Chaub. ex Fauché) Hand.-Mazz.	iN65_loli969	MEL 1591969	AUSG1097-10	N	N	Y	N	Y	Y
Lolium loliaceum (Bory & Chaub. ex Fauché) Hand.-Mazz.	iN66_loli323	MEL 534323	AUSG1098-10	Y	Y	Y	Y	Y	Y
Lolium multiflorum Lam.	iN67_mult681	MEL 612681	AUSG1099-10	Y	Y	Y	Y	Y	Y
Lolium multiflorum Lam.	iN68_mult113	MEL 2035113	AUSG1100-10	Y	Y	Y	Y	Y	Y
Lolium multiflorum Lam.	iN69_mult262	MEL 2014262	AUSG1101-10	Y	Y	Y	Y	Y	Y
Lolium perenne L.	iN71_pere323	MEL 528323	AUSG1103-10	Y	Y	Y	Y	Y	Y
Lolium perenne L.	iN72_pere634	MEL 2132634	AUSG1104-10	Y	Y	Y	Y	Y	Y
Lolium perenne L.	iN73_pere707	MEL 2238707	AUSG1105-10	Y	Y	Y	Y	Y	Y
Lolium perenne L.	iN74_pere939	MEL 1589939	AUSG1106-10	Y	Y	Y	Y	Y	Y
Lolium perenne L.	iN75_pere096	MEL 597096	AUSG1107-10	Y	N	Y	Y	Y	Y
Lolium perenne X *multiflorum*	iN76_pere256	MEL 1589256	AUSG1108-10	Y	Y	Y	Y	Y	Y
Lolium perenne X *multiflorum*	iN77_pere520	MEL 264520	AUSG1109-10	Y	Y	N	Y	Y	Y
Lolium perenne X *rigidium*	iN78_pere389	MEL 2052389	AUSG1110-10	Y	Y	Y	Y	Y	Y
Lolium perenne X *rigidium*	iN79_pere938	MEL 696938	AUSG1111-10	Y	Y	N	Y	Y	Y

Taxon	Individual reference code	Herbarium accession number[a]	BOLD reference number	*rbc*L	*mat*K	ITS	Chloroplast (*rbc*L+*mat*K)	Combined (*rbc*L+*mat* K+ITS)	Image
Lolium rigidium Gaudin	iAF75_rigi028	MEL 2377028	AUSG2151-14	Y	Y	Y	Y	Y	Y
Lolium rigidium Gaudin	iN81_rigi597	MEL 1598597	AUSG1113-10	Y	Y	Y	Y	Y	Y
Lolium rigidium Gaudin	iN84_rigi013	MEL 2015013	AUSG1116-10	Y	Y	Y	Y	Y	Y
Lolium rigidium Gaudin	iN85_rigi424	MEL 1603424	AUSG1117-10	N	Y	N	Y	Y	Y
Lolium temulentum var. *arvense (With.) Lilj.*	iAE76_tema164	HO 537164	AUSG2230-14	Y	Y	Y	Y	Y	Y
Parapholis incurva (L.) C.E.Hubb.	iAD44_incu693	MEL 2061693	AUSG1164-10	Y	Y	Y	Y	Y	Y
Parapholis incurva (L.) C.E.Hubb.	iAD45_incu419	MEL 294419	AUSG1165-10	Y	Y	Y	Y	Y	Y
Parapholis incurva (L.) C.E.Hubb.	iAD46_incu061	MEL 2302061	AUSG1166-10	Y	Y	Y	Y	Y	Y
Parapholis incurva (L.) C.E.Hubb.	iAD47_incu434	MEL 594434	AUSG1167-10	Y	N	Y	Y	Y	Y
Parapholis incurva (L.) C.E.Hubb.	iAG91_incu032	MEL 2377032	AUSG2153-14	Y	Y	Y	Y	Y	Y
Parapholis incurva (L.) C.E.Hubb	iAD50_incu605	MEL 566605	AUSG1170-10	Y	Y	Y	Y	Y	Y
Parapholis strigosa (Dumort.) C.E.Hubb.	iAD48_stri132	MEL 2024132	AUSG1168-10	Y	Y	Y	Y	Y	Y
Parapholis strigosa (Dumort.) C.E.Hubb.	iAD49_stri723	MEL 579723	AUSG1169-10	Y	Y	N	Y	Y	Y
Parapholis strigosa (Dumort.) C.E.Hubb.	iAE18_stri929	MEL 2355929	AUSG2084-14	Y	Y	Y	Y	Y	Y (proxy)
Poa aff. *crassicaudex*	iAG78_crasaff086	MEL 2377086	AUSG2168-14	Y	Y	Y	Y	Y	Y
Poa aff. *crassicaudex*	iAG79_crasaff048	MEL 2377048	AUSG2157-14	Y	Y	Y	Y	Y	Y
Poa aff. *labillardierei*	iAG81_labaff080	MEL 2377080	AUSG2162-14	Y	Y	Y	Y	Y	Y
Poa aff. *labillardierei*	iAG82_labaff079	MEL 2377079	AUSG2161-14	Y	Y	Y	Y	Y	Y
Poa aff. *sieberiana*	iAE45_sieb			Y	Y	Y	Y	Y	
Poa aff. *tenera*	iAG77_teneaff085	MEL 2360851	AUSG2138-14	Y	Y	Y	Y	Y	Y
Poa aff. *tenera*	iAG80_teneaff078	MEL 2377078	AUSG2160-14	Y	Y	Y	Y	Y	Y
Poa affinis R.Br.	iAE24_affi570	MEL 293570	AUSG1999-14	Y	Y	Y	Y	Y	Y
Poa affinis R.Br.	iN87_affi957	MEL 2132957	AUSG1119-10	Y	Y	Y	Y	Y	Y
Poa amplexicaulis C.M.Weiller & Stajsic	iAE20_ampl931	MEL 2355931	AUSG2086-14	Y	Y	Y	Y	Y	Y
Poa amplexicaulis C.M.Weiller & Stajsic	iAG01_ampl900	MEL 2017900	AUSG2037-14	Y	Y	Y	Y	Y	Y
Poa amplexicaulis C.M.Weiller & Stajsic	iN88_ampl430	MEL 2308430	AUSG1120-10	Y	Y	Y	Y	Y	Y
Poa annua L.	iAG03_annu223	MEL 1526223	AUSG2011-14	Y	Y	Y	Y	Y	Y
Poa annua L.	iN90_annu333	MEL 528333	AUSG1122-10	Y	Y	N	Y	Y	Y
Poa annua L.	iN91_annu204	MEL 2195204	AUSG1123-10	Y	Y	Y	Y	Y	Y
Poa annua L.	iN92_annu989	MEL 1530989	AUSG1124-10	Y	Y	N	Y	Y	Y
Poa annua L.	iN93_annu122	MEL 2020122	AUSG1125-10	Y	Y	Y	Y	Y	Y
Poa billardierei (Spreng.) St.-Yves	iAG62_bill017	MEL 2377017	AUSG2145-14	Y	Y	Y	Y	Y	Y
Poa billardierei (Spreng.) St.-Yves	iAG63_bill022	MEL 2377022	AUSG2147-14	Y	Y	Y	Y	Y	Y
Poa billardierei (Spreng.) St.-Yves	iN19_bill735	MEL 1578735	AUSG1065-10	Y	Y	Y	Y	Y	Y
Poa billardierei (Spreng.) St.-Yves	iN20_bill746	MEL 2269746	AUSG1066-10	Y	Y	Y	Y	Y	Y
Poa billardierei (Spreng.) St.-Yves	iN21_bill710	MEL 301710		Y	N	Y	Y	Y	N
Poa bulbosa L.	iAG04_bulb789	MEL 1591789	AUSG2025-14	Y	Y	Y	Y	Y	Y
Poa bulbosa L.	iAG05_bulb152	MEL 2024152	AUSG2041-14	Y	Y	Y	Y	Y	Y
Poa bulbosa L	iAG06_bulb700	MEL 2025700	AUSG2043-14	Y	N	Y	Y	Y	Y
Poa bulbosa L.	iAG07_bulb424	MEL 1557424	AUSG2017-14	Y	Y	Y	Y	Y	Y
Poa bulbosa L.	iN95_bulb151	MEL 2313151	AUSG1127-10	Y	Y	Y	Y	Y	Y
Poa bulbosa L. var. *bulbosa*	iN94_bulbb699	MEL 2321699	AUSG1126-10	Y	Y	Y	Y	Y	Y
Poa cheelii Vickery	iAE25_chee923	MEL 1536923	AUSG2013-14	Y	Y	N	Y	Y	Y
Poa cheelii Vickery	iAE75_chee467	BRI AQ0408056	AUSG2181-14	Y	Y	Y	Y	Y	Y
Poa cheelii Vickery	iN96_chee274	MEL 690274	AUSG1128-10	Y	Y	Y	Y	Y	Y

Taxon	Individual reference code	Herbarium accession number[a]	BOLD reference number	*rbc*L	*mat*K	ITS	Chloroplast (*rbc*L+*mat*K)	Combined (*rbc*L+*mat*K+ITS)	Image
Poa clelandii Vickery	iAC01_clee454	MEL 1586454	AUSG951-10	Y	Y	Y	Y	Y	Y
Poa clelandii Vickery	iAC02_clee139	MEL 2291139	AUSG952-10	Y	Y	Y	Y	Y	Y
Poa clelandii Vickery	iAC03_clee706	MEL 290706	AUSG953-10	Y	Y	Y	Y	Y	Y
Poa clelandii Vickery	iAE05_clee083	MEL 2377083	AUSG2165-14	Y	Y	Y	Y	Y	Y
Poa clelandii Vickery	iAE06_clee093	MEL 2377093	AUSG2169-14	Y	Y	Y	Y	Y	Y
Poa clelandii Vickery	iAE11_clee919	MEL 2355919	AUSG2074-14	Y	Y	Y	Y	Y	Y
Poa clivicola Vickery	iAC04_cliv342	MEL 1617342	AUSG954-10	Y	Y	Y	Y	Y	Y
Poa clivicola Vickery	iAC05_cliv071	MEL 2013071	AUSG955-10	Y	Y	Y	Y	Y	Y
Poa clivicola Vickery	iAC06_cliv892	MEL 268892	AUSG956-10	Y	Y	N	Y	Y	Y
Poa clivicola Vickery	iAG09_cliv429	MEL 1581429	AUSG2021-14	Y	Y	Y	Y	Y	Y
Poa clivicola Vickery	iAG10_cliv388	MEL 2357388	AUSG2114-14	Y	Y	Y	Y	Y	Y
Poa compressa L.	iAC07_comp027	MEL 2296027	AUSG957-10	Y	Y	Y	Y	Y	Y
Poa cookii (Hook.f.) Hook.f.	iAC08_cook900	MEL 39900	AUSG958-10	Y	Y	Y	Y	Y	Y
Poa cookii (Hook.f.) Hook.f.	iAH71_cook564	CBG 8910564	AUSG2217-14	Y	N	Y	Y	Y	Y
Poa cookii (Hook.f.) Hook.f.	iAH72_cook118	CBG 9304118	AUSG2221-14	Y	Y	Y	Y	Y	Y
Poa cookii (Hook.f.) Hook.f.	iAH73_cook638	CBG 9303638	AUSG2219-14	Y	Y	Y	Y	Y	Y
Poa costiniana Vickery	iAC09_cons362	MEL 294362	AUSG959-10	Y	Y	Y	Y	Y	Y
Poa costiniana Vickery	iAC10_cons209	MEL 2013209	AUSG960-10	Y	Y	Y	Y	Y	Y
Poa costiniana Vickery	iAC11_cons784	MEL 247784	AUSG961-10	Y	Y	Y	Y	Y	Y
Poa costiniana Vickery	iAE23_cost934	MEL 2355934	AUSG2089-14	Y	Y	Y	Y	Y	Y
Poa costiniana Vickery	iAG08_cost718	MEL 0629718	AUSG2004-14	Y	Y	Y	Y	Y	Y
Poa costiniana Vickery	iAG12_cost099	MEL 2340099	AUSG2072-14	Y	Y	Y	Y	Y	Y
Poa crassicaudex Vickery	iAC12_cras003	MEL 619003	AUSG962-10	Y	Y	Y	Y	Y	Y
Poa crassicaudex Vickery	iAE26_cras214	MEL 696214	AUSG2010-14	Y	N	Y	Y	Y	Y
Poa drummondiana Nees.	iAC13_drum177	MEL 1554177	AUSG963-10	Y	Y	Y	Y	Y	Y
Poa drummondiana Nees.	iAC14_drum437	MEL 1599437	AUSG964-10	Y	Y	Y	Y	Y	Y
Poa drummondiana Nees.	iAG13_drum090	MEL 0576090	AUSG2000-14	Y	Y	Y	Y	Y	Y
Poa drummondiana Nees.	iAG14_drum930	MEL 1598930	AUSG2030-14	Y	Y	Y	Y	Y	Y
Poa drummondiana Nees.	iAG60_drum012	MEL 2377012	AUSG2141-14	Y	Y	Y	Y	Y	Y
Poa drummondiana Nees.	iAG61_drum016	MEL 2377016	AUSG2144-14	Y	Y	Y	Y	Y	Y
Poa ensiformis Vickery	iAC15_ensi062	MEL 620062	AUSG965-10	N	Y	Y	Y	Y	Y
Poa ensiformis Vickery	iAC16_ensi787	MEL 2323787	AUSG966-10	Y	Y	Y	Y	Y	Y
Poa ensiformis Vickery	iAC17_ensi398	MEL 2296398	AUSG967-10	Y	Y	Y	Y	Y	Y
Poa ensiformis Vickery	iAE01_ensi950	MEL 2386950		Y	Y	Y	Y	Y	N
Poa ensiformis Vickery	iAG11_ensi348	MEL 2338348	AUSG2071-14	Y	Y	Y	Y	Y	Y
Poa ensiformis Vickery	iAG58_ensi938	MEL 2355938	AUSG2092-14	Y	Y	Y	Y	Y	Y
Poa fawcettiae Vickery	iAC18_fawc998	MEL 2339998	AUSG968-10	Y	Y	Y	Y	Y	Y
Poa fawcettiae Vickery	iAC19_fawc932	MEL 2280932	AUSG969-10	Y	Y	Y	Y	Y	Y
Poa fawcettiae Vickery	iAC20_fawc201	MEL 695201	AUSG970-10	Y	Y	Y	Y	Y	Y
Poa fawcettiae Vickery	iAG16_fawc436	MEL 0612436	AUSG2001-14	Y	Y	Y	Y	Y	Y
Poa fawcettiae Vickery	iAG17_fawc242	MEL 2338242	AUSG2070-14	Y	Y	Y	Y	Y	Y
Poa fawcettiae Vickery	iAG56_fawc935	MEL 2355935	AUSG2090-14	Y	Y	Y	Y	Y	Y
Poa fax J.H.Willis & Court	iAC21_fax869	MEL 501869	AUSG971-10	Y	Y	Y	Y	Y	Y
Poa fax J.H.Willis & Court	iAC22_fax093	MEL 2060093	AUSG972-10	Y	Y	Y	Y	Y	Y
Poa fax J.H.Willis & Court	iAE59_fax579	MEL 1592579	AUSG2028-14	N	N	Y	N	Y	Y

Taxon	Individual reference code	Herbarium accession number[a]	BOLD reference number	*rbc*L	*mat*K	ITS	Chloroplast (*rbc*L+*mat*K)	Combined (*rbc*L+*mat* K+ITS)	Image
Poa fax J.H.Willis & Court	iAE60_fax187	MEL 1560187	AUSG2019-14	Y	N	Y	Y	Y	Y
Poa fax J.H.Willis & Court	iAG15_fax603	MEL 0688603	AUSG2008-14	Y	Y	Y	Y	Y	Y
Poa foliosa (Hook.f.) Hook.f.	iAC23_foli853	MEL 2274853	AUSG973-10	Y	Y	Y	Y	Y	Y
Poa foliosa (Hook.f.) Hook.f.	iAC24_foli943	MEL 2311943	AUSG974-10	Y	Y	Y	Y	Y	Y
Poa foliosa (Hook.f.) Hook.f.	iAH60_foli351	CBG 8803351	AUSG2216-14	Y	Y	Y	Y	Y	Y
Poa foliosa (Hook.f.) Hook.f.	iAH61_foli928	CBG 8800928	AUSG2214-14	Y	Y	Y	Y	Y	Y
Poa foliosa (Hook.f.) Hook.f.	iAH62_foli096	CBG 9304096	AUSG2220-14	Y	Y	Y	Y	Y	Y
Poa foliosa (Hook.f.) Hook.f.	iAH63_foli071	CANB 379307	AUSG2193-14	Y	Y	Y	Y	Y	Y
Poa foliosa (Hook.f.) Hook.f.	iAH64_foli576	CBG 8910576	AUSG2218-14	Y	N	Y	Y	Y	Y
Poa foliosa (Hook.f.) Hook.f.	iAH70_cook929	CBG 8800929	AUSG2215-14	Y	N	Y	Y	Y	Y
Poa fordeana F.Muell.	iAC25_ford246	MEL 2199246	AUSG975-10	Y	Y	Y	Y	Y	Y
Poa fordeana F.Muell.	iAC26_ford519	MEL 1589519	AUSG976-10	Y	Y	Y	Y	Y	Y
Poa fordeana F.Muell.	iAG18_ford110	MEL 2357110	AUSG2110-14	Y	Y	Y	Y	Y	Y
Poa fordeana F.Muell.	iAG19_ford880	MEL 2352880	AUSG2073-14	Y	Y	Y	Y	Y	Y
Poa fordeana F.Muell.	iAG20_ford172	MEL 2358172	AUSG2116-14	Y	Y	Y	Y	Y	Y
Poa gunnii Vickery	iAC27_gunn793	MEL 2309793	AUSG977-10	Y	Y	Y	Y	Y	Y
Poa gunnii Vickery	iAC28_gunn762	MEL 652762	AUSG978-10	Y	Y	Y	Y	Y	Y
Poa gunnii Vickery	iAG21_gunn883	MEL 626683	AUSG2003-14	Y	Y	Y	Y	Y	Y
Poa gunnii Vickery	iAG22_gunn044	MEL 0663044	AUSG2005-14	Y	Y	Y	Y	Y	Y
Poa gunnii Vickery	iAG23_gunn915	MEL 1577915	AUSG2020-14	Y	Y	Y	Y	Y	Y
Poa gunnii Vickery	iAG24_gunn644	MEL 1617644	AUSG2033-14	Y	Y	Y	Y	Y	Y
Poa halmaturina J.M.Black	iAE61_halm714	HO 69714	AUSG2235-14	Y	Y	Y	Y	Y	Y
Poa hamiltonii Kirk	iAC29_hami903	MEL 39903	AUSG979-10	Y	Y	Y	Y	Y	Y
Poa helmsii Vickery	iAC30_helm286	MEL 2234286	AUSG980-10	Y	Y	Y	Y	Y	Y
Poa helmsii Vickery	iAC31_helm630	MEL 2296630	AUSG981-10	Y	Y	Y	Y	Y	Y
Poa helmsii Vickery	iAC32_helm871	MEL 2325871	AUSG982-10	Y	Y	Y	Y	Y	Y
Poa helmsii Vickery	iAG25_helm184	MEL 677184	AUSG2006-14	Y	Y	Y	Y	Y	Y
Poa helmsii Vickery	iAG26_helm051	MEL 2213051	AUSG2053-14	Y	Y	Y	Y	Y	Y
Poa hiemata Vickery	iAC33_hiem287	MEL 1582287	AUSG983-10	Y	N	Y	Y	Y	Y
Poa hiemata Vickery	iAC34_hiem617	MEL 693617	AUSG984-10	Y	Y	Y	Y	Y	Y
Poa hiemata Vickery	iAC35_hiem322	MEL 2046322	AUSG985-10	Y	Y	Y	Y	Y	Y
Poa hiemata Vickery	iAE22_hiem933	MEL 2355933	AUSG2088-14	Y	Y	Y	Y	Y	Y
Poa hiemata Vickery	iAG57_hiem936	MEL 2355936	AUSG2091-14	Y	Y	Y	Y	Y	Y
Poa hiemata Vickery	iAG83_hiem941	MEL 2355941	AUSG2095-14	Y	Y	Y	Y	Y	Y
Poa homomalla Nees.	iAE30_homo957	MEL 2355957	AUSG2102-14	N	Y	Y	Y	Y	Y
Poa homomalla Nees.	iAE31_homo959	MEL 2355959	AUSG2104-14	Y	Y	Y	Y	Y	Y
Poa homomalla Nees.	iAE32_homo960	MEL 2355960	AUSG2105-14	Y	Y	Y	Y	Y	Y
Poa homomalla Nees.	iAG64_homo023	MEL 2377023	AUSG2148-14	Y	Y	Y	Y	Y	Y
Poa hookeri Vickery	iAC36_hook099	MEL 2123099	AUSG986-10	Y	Y	Y	Y	Y	Y
Poa hookeri Vickery	iAC37_hook792	MEL 2323792	AUSG987-10	Y	Y	Y	Y	Y	Y
Poa hookeri Vickery	iAC38_hook824	MEL 2323824	AUSG988-10	Y	Y	Y	Y	Y	Y
Poa hookeri Vickery	iAE51_hook383	MEL 2357383	AUSG2113-14	Y	Y	Y	Y	Y	Y
Poa hookeri Vickery	iAE52_hook209	MEL 2314209	AUSG2061-14	Y	Y	Y	Y	Y	Y
Poa hothamensis var. *parviflora* N.G.Walsh	iAC41_hothp794	MEL 1597794	AUSG991-10	Y	Y	Y	Y	Y	Y
Poa hothamensis var. *parviflora* N.G.Walsh	iAC42_hothp344	MEL 1564344	AUSG992-10	Y	Y	Y	Y	Y	Y

Taxon	Individual reference code	Herbarium accession number[a]	BOLD reference number	*rbc*L	*mat*K	ITS	Chloroplast (*rbc*L+*mat*K)	Combined (*rbc*L+*mat*K+ITS)	Image
Poa hothamensis var. *parviflora* N.G.Walsh	iAE53_hothp076	MEL 113076	AUSG1995-14	Y	Y	Y	Y	Y	Y
Poa hothamensis var. *parviflora* N.G.Walsh	iAE54_hothp812	MEL 1556812	AUSG2016-14	Y	Y	Y	Y	Y	Y
Poa hothamensis var. *parviflora* N.G.Walsh	iAE55_hothp844	MEL 1557844	AUSG2018-14	Y	Y	Y	Y	Y	Y
Poa hothamensis Vickery var. *hothamensis*	iAC39_hothh845	MEL 2275845	AUSG989-10	Y	Y	Y	Y	Y	Y
Poa hothamensis Vickery var. *hothamensis*	iAC40_hothh980	MEL 2014980	AUSG990-10	Y	Y	Y	Y	Y	Y
Poa hothamensis Vickery var. *hothamensis*	iAE56_hothh092	MEL 1545092	AUSG2015-14	Y	Y	N	Y	Y	Y
Poa hothamensis Vickery var. *hothamensis*	iAE57_hothh923	MEL 2012923	AUSG2035-14	Y	N	Y	Y	Y	Y
Poa hothamensis Vickery var. *hothamensis*	iAE58_hothh150	MEL 1592150	AUSG2026-14	Y	Y	Y	Y	Y	Y
Poa induta Vickery	iAC43_indu408	MEL 2253408	AUSG993-10	Y	Y	Y	Y	Y	Y
Poa induta Vickery	iAE02_indu	MEL 2360858	AUSG2139-14	Y	Y	Y	Y	Y	Y
Poa induta Vickery	iAH65_indu255	CBG 8605255	AUSG2213-14	Y	Y	Y	Y	Y	Y
Poa induta Vickery	iAH66_indu917	CANB 645917	AUSG2205-14	Y	Y	Y	Y	Y	Y
Poa induta Vickery	iAH67_indu061	CANB 804061	AUSG2211-14	Y	Y	Y	Y	Y	Y
Poa induta Vickery	iAH68_indu200	CANB 647200	AUSG2206-14	Y	Y	Y	Y	Y	Y
Poa induta Vickery	iAH69_indu161	CANB 526161	AUSG2199-14	Y	N	Y	Y	Y	Y
Poa infirma Kunth	iAC44_infi528	MEL 2084528	AUSG994-10	Y	Y	Y	Y	Y	Y
Poa infirma Kunth	iAC46_infi801	MEL 2069801		Y	Y	Y	Y	Y	Y
Poa infirma Kunth	iAG27_infi059	MEL 2012059	AUSG2034-14	Y	Y	Y	Y	Y	Y
Poa infirma Kunth	iAG28_infi123	MEL 2020123	AUSG2039-14	Y	Y	Y	Y	Y	Y
Poa jugicola D.I.Morris	iAE27_jugi609	MEL 1584609	AUSG2022-14	Y	Y	Y	Y	Y	Y
Poa jugicola D.I.Morris	iAG52_jugi382	HO 109382	AUSG2222-14	Y	Y	Y	Y	Y	Y
Poa jugicola D.I.Morris	iAG53_jugi178	HO 126178	AUSG2223-14	Y	Y	Y	Y	Y	Y
Poa jugicola D.I.Morris	iAG54_jugi271	HO 317271	AUSG2225-14	Y	Y	Y	Y	Y	Y
Poa jugicola D.I.Morris	iAG55_jugi579	HO 144579	AUSG2224-14	Y	Y	Y	Y	Y	Y
Poa labillardierei "Volcanic Plains"	iAG65_labv924	MEL 2355924	AUSG2079-14	Y	Y	Y	Y	Y	Y
Poa labillardierei "Volcanic Plains"	iAG66_labv926	MEL 2355926	AUSG2081-14	Y	Y	Y	Y	Y	Y
Poa labillardierei "Volcanic Plains"	iAG67_labv060	MEL 2377060	AUSG2158-14	Y	Y	Y	Y	Y	Y
Poa labillardierei "Volcanic Plains"	iAG67_labv060	MEL 2377060		Y	Y	Y	Y	Y	N
Poa labillardierei Steud. var. *labillardierei*	iAC50_labl620	MEL 253620	AUSG1000-10	Y	Y	Y	Y	Y	Y
Poa labillardierei Steud. var. *labillardierei*	iAC51_labl896	MEL 2323896	AUSG1001-10	Y	Y	Y	Y	Y	Y
Poa labillardierei Steud. var. *labillardierei*	iAC52_labl192	MEL 2312192	AUSG1002-10	Y	Y	Y	Y	Y	Y
Poa labillardierei Steud. var. *labillardierei*	iAE04_labl084	MEL 2377084	AUSG2166-14	Y	Y	Y	Y	Y	Y
Poa labillardierei Steud. var. *labillardierei*	iAE19_labl930	MEL 2355930	AUSG2085-14	Y	Y	Y	Y	Y	Y
Poa labillardierei Steud. var. *labillardierei*	iAE42_labl939	MEL 2355939	AUSG2093-14	Y	Y	Y	Y	Y	Y
Poa labillardierei Steud. var. *labillardierei*	iAE43_labl394	MEL 2358694A	AUSG2117-14	Y	Y	Y	Y	Y	Y
Poa labillardierei var. *acris* Vickery	iAC48_laba214	MEL 2312214	AUSG998-10	Y	Y	Y	Y	Y	Y
Poa labillardierei var. *acris* Vickery	iAC49_laba741	MEL 722741	AUSG999-10	Y	Y	Y	Y	Y	Y
Poa litorosa Cheeseman	iAC53_lito378	MEL 306378	AUSG1003-10	Y	Y	Y	Y	Y	Y
Poa litorosa Cheeseman	iAH84_lito230	CANB 541230	AUSG2202-14	Y	Y	Y	Y	Y	Y
Poa litorosa Cheeseman	iAH85_lito030	CANB 9304030	AUSG2212-14	Y	Y	Y	Y	Y	Y
Poa lowanensis N.G.Walsh	iAC54_lowa394	MEL 2212394	AUSG1004-10	Y	Y	Y	Y	Y	Y
Poa lowanensis N.G.Walsh	iAC55_lowa028	MEL 2010028	AUSG1005-10	Y	Y	Y	Y	Y	Y
Poa lowanensis N.G.Walsh	iAG29_lowe434	MEL 1599434	AUSG2031-14	Y	Y	Y	Y	Y	Y
Poa lowanensis N.G.Walsh	iAG30_lowe440	MEL 1599440	AUSG2032-14	Y	Y	Y	Y	Y	Y
Poa lowanensis N.G.Walsh	iAG31_lowe766	MEL 2323766	AUSG2067-14	Y	Y	Y	Y	Y	Y

Taxon	Individual reference code	Herbarium accession number[a]	BOLD reference number	*rbc*L	*mat*K	ITS	Chloroplast (*rbc*L+*mat*K)	Combined (*rbc*L+*mat*K+ITS)	Image
Poa meionectes Vickery	iAC56_meio290	MEL 1556290	AUSG1006-10	Y	Y	Y	Y	Y	Y
Poa meionectes Vickery	iAC57_meio401	MEL 2045401	AUSG1007-10	Y	Y	Y	Y	Y	Y
Poa meionectes Vickery	iAG32_meio933	MEL 0683933	AUSG2007-14	Y	Y	Y	Y	Y	Y
Poa meionectes Vickery	iAG33_meio310	MEL 0690310	AUSG2009-14	Y	Y	Y	Y	Y	Y
Poa meionectes Vickery	iAG34_meio917	MEL 1597917	AUSG2029-14	Y	Y	Y	Y	Y	Y
Poa mollis Vickery	iAE28_moll799	MEL 112799	AUSG1994-14	Y	Y	Y	Y	Y	Y
Poa mollis Vickery	iAG49_moll809	HO 534809	AUSG2229-14	Y	Y	Y	Y	Y	Y
Poa mollis Vickery	iAG50_moll988	HO 554988	AUSG2233-14	Y	Y	Y	Y	Y	Y
Poa mollis Vickery	iAG51_moll406	HO 560406	AUSG2234-14	Y	Y	Y	Y	Y	Y
Poa morrisii Vickery	iAC58_morr400	MEL 2059400	AUSG1008-10	Y	Y	Y	Y	Y	Y
Poa morrisii Vickery	iAC59_morr525	MEL 2012525	AUSG1009-10	Y	Y	Y	Y	Y	Y
Poa morrisii Vickery	iAC60_morr028	MEL 2299028	AUSG1010-10	Y	Y	Y	Y	Y	Y
Poa morrisii Vickery	iAG35_morr247	MEL 2051247	AUSG2048-14	Y	N	Y	Y	Y	Y
Poa morrisii Vickery	iAG59_morr944	MEL 2355944	AUSG2097-14	Y	Y	Y	Y	Y	Y
Poa morrisii Vickery	iAG84_morr942	MEL 2355942	AUSG2096-14	Y	Y	Y	Y	Y	Y
Poa orba N.G.Walsh	iAC61_orba483	MEL 2212483	AUSG1011-10	Y	Y	Y	Y	Y	Y
Poa orba N.G.Walsh	iAE29_orba762	MEL 2323762	AUSG2065-14	Y	Y	Y	Y	Y	Y
Poa orthoclada N.G.Walsh	iAC62_orth002	MEL 2296022	AUSG1012-10	Y	Y	Y	Y	Y	Y
Poa orthoclada N.G.Walsh	iAC63_orth158	MEL 2054158	AUSG1013-10	Y	Y	Y	Y	Y	Y
Poa orthoclada N.G.Walsh	iAC64_orth826	MEL 2283826	AUSG1014-10	Y	Y	Y	Y	Y	Y
Poa orthoclada N.G.Walsh	iAG38_orth822	MEL 2275822	AUSG2057-14	Y	Y	Y	Y	Y	Y
Poa orthoclada N.G.Walsh	iAG39_orth761	MEL 2323761	AUSG2064-14	Y	Y	Y	Y	Y	Y
Poa orthoclada N.G.Walsh	iAG40_orth373	MEL 2357373	AUSG2112-14	Y	Y	Y	Y	Y	Y
Poa petrophila Vickery	iAC65_petr290	MEL 1535290	AUSG1015-10	Y	Y	Y	Y	Y	Y
Poa petrophila Vickery	iAC66_petr917	MEL 524917	AUSG1016-10	Y	Y	Y	Y	Y	Y
Poa petrophila Vickery	iAG36_petr933	MEL 2325933	AUSG2068-14	Y	Y	Y	Y	Y	Y
Poa phillipsiana Vickery	iAE44_phil940	MEL 2355940	AUSG2094-14	Y	Y	Y	Y	Y	Y
Poa phillipsiana Vickery	iAE46_phil049	MEL 222049	AUSG1996-14	Y	Y	Y	Y	Y	Y
Poa phillipsiana Vickery	iAE47_phil997	MEL 2012997	AUSG2036-14	Y	Y	Y	Y	Y	Y
Poa phillipsiana Vickery	iN22_phil566	MEL 2129566	AUSG1068-10	Y	Y	Y	Y	Y	Y
Poa phillipsiana Vickery	iN23_phil584	MEL 2296584	AUSG1069-10	Y	Y	Y	Y	Y	Y
Poa phillipsiana Vickery	iN24_phil838	MEL 2275838	AUSG1070-10	Y	Y	Y	Y	Y	Y
Poa physoclina N.G.Walsh	iAC67_phys592	MEL 2328592	AUSG1017-10	Y	Y	Y	Y	Y	Y
Poa physoclina N.G.Walsh	iAC68_phys894	MEL 2329894	AUSG1018-10	Y	Y	Y	Y	Y	Y
Poa physoclina N.G.Walsh	iAE16_phys927	MEL 2355927	AUSG2082-14	Y	Y	Y	Y	Y	Y
Poa physoclina N.G.Walsh	iAG41_phys304	MEL 2314304	AUSG2062-14	Y	Y	Y	Y	Y	Y
Poa poiformis (Labill.) Druce var. *poiformis*	iAC69_poifp392	MEL 2031392	AUSG1019-10	Y	Y	Y	Y	Y	Y
Poa poiformis (Labill.) Druce var. *poiformis*	iAC70_poifp500	MEL 2262500	AUSG1020-10	Y	Y	Y	Y	Y	Y
Poa poiformis (Labill.) Druce var. *poiformis*	iAE13_poifp922	MEL 2355922	AUSG2077-14	Y	Y	Y	Y	Y	Y
Poa poiformis (Labill.) Druce var. *poiformis*	iAE33_poifp963	MEL 2355963	AUSG2107-14	Y	Y	Y	Y	Y	Y
Poa poiformis (Labill.) Druce var. *poiformis*	iAE34_poifp018	MEL 2377018	AUSG2146-14	Y	Y	Y	Y	Y	Y
Poa poiformis compact	iAG75_poif921	MEL 2355921	AUSG2076-14	Y	Y	Y	Y	Y	Y
Poa poiformis var. *ramifer* D.I.Morris	iAC71_poifr499	MEL 2092494	AUSG1021-10	Y	Y	Y	Y	Y	Y
Poa poiformis var. *ramifer* D.I.Morris	iAC72_poifr794	MEL 2309794	AUSG1022-10	Y	Y	Y	Y	Y	Y
Poa poiformis var. *ramifer* D.I.Morris	iAE12_poifr920	MEL 2355920	AUSG2075-14	Y	Y	Y	Y	Y	Y

Taxon	Individual reference code	Herbarium accession number[a]	BOLD reference number	*rbc*L	*mat*K	ITS	Chloroplast (*rbc*L+*mat*K)	Combined (*rbc*L+*mat*K+ITS)	Image
Poa poiformis var. *ramifer* D.I.Morris	iAE35_poifr962	MEL 2355962	AUSG2106-14	Y	Y	Y	Y	Y	Y
Poa poiformis var. *ramifer* D.I.Morris	iAE36_poifr964	MEL 2355964	AUSG2108-14	Y	Y	Y	Y	Y	Y
Poa porphyroclados Nees.	iAC73_porp212	MEL 268212	AUSG1023-10	Y	Y	Y	Y	Y	Y
Poa porphyroclados Nees.	iAE37_porp958	MEL 2355958	AUSG2103-14	Y	Y	Y	Y	Y	Y
Poa porphyroclados Nees.	iAE38_porp013	MEL 2377013	AUSG2142-14	Y	Y	Y	Y	Y	Y
Poa porphyroclados Nees.	iAE39_porp014	MEL 2377014	AUSG2143-14	Y	Y	Y	Y	Y	Y
Poa porphyroclados Nees.	iAE40_porp024	MEL 2377024	AUSG2149-14	Y	Y	Y	Y	Y	Y
Poa porphyroclados Nees.	iAG85_porp025	MEL 2377025	AUSG2150-14	Y	Y	Y	Y	Y	Y
Poa pratensis L.	iAC74_prat128	MEL 693128	AUSG1024-10	Y	Y	Y	Y	Y	Y
Poa pratensis L.	iAC75_prat781	MEL 2291781	AUSG1025-10	Y	Y	Y	Y	Y	Y
Poa pratensis L.	iAC76_prat027	MEL 1580027	AUSG1026-10	Y	Y	Y	Y	Y	Y
Poa pratensis L.	iAC77_prat115	MEL 1503115	AUSG1027-10	Y	Y	Y	Y	Y	Y
Poa pratensis L.	iAG37_prat143	MEL 2024143	AUSG2040-14	Y	N	Y	Y	Y	Y
Poa rodwayi Vickery	iAC80_rodw221	MEL 696221	AUSG1030-10	Y	Y	Y	Y	Y	Y
Poa rodwayi Vickery	iAC81_rodw897	MEL 2323897	AUSG1031-10	Y	Y	Y	Y	Y	Y
Poa rodwayi Vickery	iAC82_rodw791	MEL 2277791	AUSG1032-10	Y	Y	Y	Y	Y	Y
Poa rodwayi Vickery	iAE15_rodw925	MEL 2355925	AUSG2080-14	Y	Y	Y	Y	Y	Y
Poa sallacustris N.G.Walsh	iAC83_sall164	MEL 2325164	AUSG1033-10	Y	Y	Y	Y	Y	Y
Poa sallacustris N.G.Walsh	iAC84_sall006	MEL 2187006	AUSG1034-10	Y	Y	Y	Y	Y	Y
Poa sallacustris N.G.Walsh	iAE14_sall923	MEL 2355923	AUSG2078-14	Y	Y	Y	Y	Y	Y
Poa sallacustris N.G.Walsh	iAG44_sall830	MEL 2089830	AUSG2051-14	Y	Y	Y	Y	Y	Y
Poa serpentum Nees	iAH82_serp634	CANB 312634	AUSG2187-14	Y	Y	Y	Y	Y	Y
Poa serpentum Nees	iAH83_serp017	CANB 257017	AUSG2185-14	Y	N	Y	Y	Y	Y
Poa sieberiana Spreng. var. *sieberiana*	iAC94_siebs381	MEL 2059381	AUSG1044-10	Y	Y	Y	Y	Y	Y
Poa sieberiana Spreng. var. *sieberiana*	iAC95_siebs829	MEL 718829	AUSG1045-10	Y	Y	Y	Y	Y	Y
Poa sieberiana Spreng. var. *sieberiana*	iAC96_siebs047	MEL 2213047	AUSG1046-10	Y	Y	Y	Y	Y	Y
Poa sieberiana Spreng. var. *sieberiana*	iN01_siebs557	MEL 2299557	AUSG1047-10	Y	N	Y	Y	Y	Y
Poa sieberiana var. *cyanophylla* Vickery	iAC88_siebc141	MEL 1617141	AUSG1038-10	Y	Y	Y	Y	Y	Y
Poa sieberiana var. *cyanophylla* Vickery	iAC89_siebc368	MEL 2325368	AUSG1039-10	Y	Y	Y	Y	Y	Y
Poa sieberiana var. *cyanophylla* Vickery	iAE48_siebc389	MEL 2357389	AUSG2115-14	Y	Y	Y	Y	Y	Y
Poa sieberiana var. *cyanophylla* Vickery	iAE49_siebc195	MEL 2312195	AUSG2060-14	Y	Y	Y	Y	Y	Y
Poa sieberiana var. *cyanophylla* Vickery	iAE50_siebc698	MEL 2123098	AUSG2052-14	Y	Y	Y	Y	Y	Y
Poa sieberiana var. *hirtella* Vickery	iAC90_siebh364	MEL 2337364	AUSG1040-10	Y	Y	Y	Y	Y	Y
Poa sieberiana var. *hirtella* Vickery	iAC91_siebh752	MEL 719752	AUSG1041-10	Y	Y	Y	Y	Y	Y
Poa sieberiana var. *hirtella* Vickery	iAC92_siebh370	MEL 2339370	AUSG1042-10	Y	Y	Y	Y	Y	Y
Poa sieberiana var. *hirtella* Vickery	iAC93_siebh568	MEL 2117568	AUSG1043-10	Y	Y	Y	Y	Y	Y
Poa sieberiana var. *hirtella* Vickery	iAE03_siebh081	MEL 2377081	AUSG2163-14	Y	Y	Y	Y	Y	Y
Poa tenera F.Muell. ex Hook.f.	iAE10_tene082	MEL 2377082	AUSG2164-14	Y	Y	Y	Y	Y	Y
Poa tenera F.Muell. ex Hook.f.	iN02_tene398	MEL 2059398	AUSG1048-10	Y	Y	Y	Y	Y	Y
Poa tenera F.Muell. ex Hook.f.	iN03_tene236	MEL 305236	AUSG1049-10	Y	Y	Y	Y	Y	Y
Poa tenera F.Muell. ex Hook.f.	iN04_tene161	MEL 2042161	AUSG1050-10	Y	Y	Y	Y	Y	Y
Poa tenera F.Muell. ex Hook.f.	iN05_tene321	MEL 2316321	AUSG1051-10	Y	N	Y	Y	Y	Y
Poa trivialis L.	iAG46_triv702	MEL 2321702	AUSG2063-14	Y	Y	Y	Y	Y	Y
Poa trivialis L.	iAG47_triv693	MEL 2331693	AUSG2069-14	Y	Y	Y	Y	Y	Y
Poa trivialis L.	iN06_triv933	MEL 2070933	AUSG1052-10	Y	Y	Y	Y	Y	Y

Taxon	Individual reference code	Herbarium accession number[a]	BOLD reference number	*rbc*L	*mat*K	ITS	Chloroplast (*rbc*L+*mat*K)	Combined (*rbc*L+*mat*K+ITS)	Image
Poa trivialis L.	iN07_triv877	MEL 2323877	AUSG1053-10	Y	Y	Y	Y	Y	Y
Poa umbricola Vickery	iAE07_umbraff085	MEL 2377085	AUSG2167-14	Y	Y	Y	Y	Y	Y
Poa umbricola Vickery	iAE09_umbr047	MEL 2377047	AUSG2156-14	Y	Y	Y	Y	Y	Y
Psilurus incurvus (Gouan) Schinz & Thell.	iAD42_incu359	MEL 1592359	AUSG1162-10	Y	Y	Y	Y	Y	Y
Psilurus incurvus (Gouan) Schinz & Thell.	iAD43_incu963	MEL 223963	AUSG1163-10	Y	Y	Y	Y	Y	Y
Psilurus incurvus (Gouan) Schinz & Thell.	iAE68_incu364	MEL 1592364	AUSG2027-14	Y	Y	Y	Y	Y	Y
Puccinellia ciliata Bor	iAE73_cili034	MEL 2377034	AUSG2155-14	Y	Y	Y	Y	Y	Y
Puccinellia ciliata Bor	iAH88_cili033	MEL 2377033	AUSG2154-14	Y	Y	Y	Y	Y	Y
Puccinellia ciliata Bor	iN09_cili920	MEL 696920	AUSG1055-10	Y	Y	Y	Y	Y	Y
Puccinellia distans (Jacq.) Parl.	iAH39_dist467	MEL 1589467	AUSG2024-14	Y	N	Y	Y	Y	Y
Puccinellia distans (Jacq.) Parl.	iAH94_dist929	MEL 2359929	AUSG2124-14	Y	Y	Y	Y	Y	Y
Puccinellia fasciculata (Torr.) E.P.Bicknell	iAH41_fasc832	MEL 225832	AUSG1998-14	Y	Y	Y	Y	Y	Y
Puccinellia fasciculata (Torr.) E.P.Bicknell	iN10_fasc915	MEL 696915	AUSG1056-10	Y	Y	Y	Y	Y	Y
Puccinellia fasciculata (Torr.) E.P.Bicknell	iN11_fasc355	MEL 2267355	AUSG1057-10	Y	Y	Y	Y	Y	Y
Puccinellia longior A.R.Williams	iAE71_long031	MEL 2377031	AUSG2152-14	Y	Y	Y	Y	Y	Y
Puccinellia perlaxa (Stapf ex N.G.Walsh) N.G.Walsh & A.R.Williams	iAE17_perl928	MEL 2355928	AUSG2083-14	Y	Y	Y	Y	Y	Y
Puccinellia perlaxa (Stapf ex N.G.Walsh) N.G.Walsh & A.R.Williams	iN12_perl624	MEL 2355928	AUSG2083-14	Y	Y	Y	Y	Y	Y
Puccinellia perlaxa (Stapf ex N.G.Walsh) N.G.Walsh & A.R.Williams	iN13_perl676	MEL 2325676	AUSG1059-10	Y	Y	Y	Y	Y	Y
Puccinellia perlaxa (Stapf ex N.G.Walsh) N.G.Walsh & A.R.Williams	iN14_perl083	MEL 2071083	AUSG1060-10	Y	Y	Y	Y	Y	Y
Puccinellia stricta (Hook.f.) C.H.Blom	iN15_stri335	MEL 2059335	AUSG1061-10	Y	Y	Y	Y	Y	Y
Puccinellia stricta (Hook.f.) C.H.Blom	iN16_stri898	MEL 2275898	AUSG1062-10	Y	Y	Y	Y	Y	Y
Puccinellia stricta (Hook.f.) C.H.Blom	iN17_stri152	MEL 2143152	AUSG1063-10	Y	Y	N	Y	Y	Y
Puccinellia stricta (Hook.f.) C.H.Blom	iN18_stri736	MEL 1581736	AUSG1064-10	Y	N	Y	Y	Y	Y
Puccinellia vassica A.R.Williams	iAE74_vass948	MEL 2355948	AUSG2098-14	Y	Y	Y	Y	Y	Y
Saxipoa saxicola (R.Br.) Soreng, L.J.Gillespie & S.W.L. Jacobs	iAC85_saxi094	MEL 2123094	AUSG1035-10	Y	Y	Y	Y	Y	Y
Saxipoa saxicola (R.Br.) Soreng, L.J.Gillespie & S.W.L. Jacobs	iAC86_saxi987	MEL 2275987	AUSG1036-10	Y	Y	Y	Y	Y	Y
Saxipoa saxicola (R.Br.) Soreng, L.J.Gillespie & S.W.L. Jacobs	iAG43_saxi764	MEL 2323764	AUSG2066-14	Y	Y	Y	Y	Y	Y
Sclerochloa dura (L.) P.Beauv.	iAD32_dura541	MEL 2016541	AUSG1152-10	Y	Y	Y	Y	Y	Y
Sclerochloa dura (L.) P.Beauv.	iAE69_dura205	MEL 1589205	AUSG2023-14	Y	Y	Y	Y	Y	Y
Sclerochloa dura (L.) P.Beauv.	iAF72_dura288	AD 99426288	AUSG2177-14	Y	N	Y	Y	Y	Y
Sclerochloa dura (L.) P.Beauv.	iAF73_dura221	AD 97009221	AUSG2174-14	Y	N	Y	Y	Y	Y
Sclerochloa dura (L.) P.Beauv.	iAF74_dura295	AD 97920295	AUSG2175-14	Y	Y	Y	Y	Y	Y
Sclerochloa dura (L.) P.Beauv.	iAH44_dura992	MEL 223992	AUSG1997-14	Y	N	Y	Y	Y	Y
Sphenopus divaricatus (Gouan) Reichb.	iAF69_diva127	AD 99551127	AUSG2178-14	Y	N	Y	Y	Y	Y
Sphenopus divaricatus (Gouan) Reichb.	iAF70_diva087	AD 99416087	AUSG2176-14	Y	Y	Y	Y	Y	Y
Sphenopus divaricatus (Gouan) Reichb.	iAF71_diva301	AD 108301	AUSG2171-14	Y	Y	Y	Y	Y	Y
Sylvipoa queenslandica (C.E.Hubb.) Soreng, L.J. Gillespie & S.W.L. Jacobs	iAC78_quee258	MEL 2164258	AUSG1028-10	Y	Y	Y	Y	Y	Y
Sylvipoa queenslandica (C.E.Hubb.) Soreng, L.J. Gillespie & S.W.L. Jacobs	iAC79_quee532	MEL 280532	AUSG1029-10	Y	Y	Y	Y	Y	Y
Vulpia bromoides L.(Gray)	iAD11_brom370	MEL 285370	AUSG1131-10	Y	N	Y	Y	Y	Y
Vulpia bromoides L.(Gray)	iAD12_brom475	MEL 2034475	AUSG1132-10	Y	Y	Y	Y	Y	Y
Vulpia bromoides L.(Gray)	iAD13_brom299	MEL 529299	AUSG1133-10	N	N	Y	N	Y	Y

Taxon	Individual reference code	Herbarium accession number[a]	BOLD reference number	*rbc*L	*mat*K	ITS	Chloroplast (*rbc*L+*mat*K)	Combined (*rbc*L+*mat*K+ITS)	Image
Vulpia ciliata (Pers.)Link	iAD15_cili385	MEL 648385	AUSG1135-10	Y	N	Y	Y	Y	Y
Vulpia fasciculata (Forssk.) Fritsch	iAD16_fasc485	MEL 1586485	AUSG1136-10	Y	Y	Y	Y	Y	Y
Vulpia fasciculata (Forssk.) Fritsch	iAD17_fasc924	MEL 2105924	AUSG1137-10	Y	Y	Y	Y	Y	Y
Vulpia muralis (Kunth) Nees	iAD19_mura647	MEL 255647	AUSG1139-10	Y	N	N	Y	N	Y
Vulpia muralis (Kunth) Nees	iAD21_mura064	MEL 1593064	AUSG1141-10	Y	N	Y	Y	Y	Y
Vulpia myuros (L.) C.C.Gmel.	iAG90_myur965	MEL 2355965	AUSG2109-14	Y	Y	Y	Y	Y	Y
Vulpia myuros (L.) C.C.Gmel. forma *myuros*	iAD25_myury891	MEL 225891	AUSG1145-10	Y	N	Y	Y	Y	Y
Vulpia myuros (L.) C.C.Gmel. forma *myuros*	iAD26_myuyr045	MEL 2010045	AUSG1146-10	Y	Y	Y	Y	Y	Y
Vulpia myuros (L.) C.C.Gmel. forma *myuros*	iAD27_myury009	MEL 2268009	AUSG1147-10	Y	Y	Y	Y	Y	Y
Vulpia myuros forma megalura (Nutt.) Stace & R.Cotton	iAD24_myure456	MEL 2141456	AUSG1144-10	Y	N	Y	Y	Y	Y
