# Supplementary material for: Testing efficacy of distance and tree-based methods for DNA barcoding of grasses (Poaceae tribe Poeae) in Australia
Source: PLoS One. 2017 Oct 30;12(10):e0186259. doi: 10.1371/journal.pone.0186259 (PMC5662090; doi:10.1371/journal.pone.0186259)
Supplement: S2 Table — (PDF) [file pone.0186259.s002.pdf]

S2 Table.

	***rbc*L**	***mat*K**	**ITS**	**Chloroplast (*rbc*L + *mat*K)**	**Combined (*rbc*L + *mat*K + ITS)**
Number of individuals sequenced (dataset A/dataset B)	391/400	354/365	383/393	395/404	399/406
Aligned length (base pairs)	555	739	718	1294	2012
Sequence length parameters: minimum/average (standard deviation)/maximum.	366/552.1(12.3)/ 555	150/678.0(110.2)/739	221/660.3(44.7)/689	555/1224.5(213.4)/1294	555/1873.5(279.2)/2012
Missing data (%)	0.2	0.3	4.8	0.7	8.2
GC content (%)	43.8	32.2	57.1	37.8	41.5
Model of evolution	K80+I	TVM+I+G	GTR+I+G	TVM+I+G	HKY+I+G
