# Supplementary material for: Testing efficacy of distance and tree-based methods for DNA barcoding of grasses (Poaceae tribe Poeae) in Australia
Source: PLoS One. 2017 Oct 30;12(10):e0186259. doi: 10.1371/journal.pone.0186259 (PMC5662090; doi:10.1371/journal.pone.0186259)
Supplement: S3 Table — BCM, Best close match; ITS, Internal transcribed spacer; NN, Nearest neighbour; TID, Threshold Identification. aMarkers for which no single threshold was optimal across the range tested and for which a default value of 0.100% was applied are indicated with an asterisk (*). bPercentages of “true” and “correct” identifications are indicated in bold. (PDF) [file pone.0186259.s003.pdf]

S3 Table.

Taxon or clade	Genetic marker	Number of genera/species	Number of individuals [**total** (minimum, **mean,** maximum)]	Specimen identification (%)			Threshold applied (% divergence)[a]
				NN (**true**/false)	BCM (**correct**/incorrect/ ambiguous/no identification)	TID (**correct**/incorrect/ ambiguous/no identification)	
**Rank**				**Genus**	**Genus**	**Genus**	**Genus**
Tribe *Poeae*	*rbc*L	18/82	391 (2,5,15)	**93.3**/6.7[b]	**43.5**/1.0/51.5/4.0	**43.5**/1.0/51.5/4.0	0.151
	*mat*K	18/79	354 (1,4,15)	**91.0**/9.0	**73.2**/2.2/16.4/8.2	**73.2**/2.2/16.4/8.2	0.101
	ITS	18/82	383 (2,5,15)	**99.5**/0.5	**97.6**/0.3/0.8/1.3	**96.6**/0.0/2.1/1.3	0.160
	*rbc*L+*mat*K	18/83	395 (2,5,15)	**93.9**/6.1	**80.8**/1.8/12.9/4.5	**80.5**/1.3/13.7/4.5	0.15
	*rbc*L+*mat*K+ITS	18/83	399 (2,5,15)	**92.0**/8.0	**87.5**/5.8/3.0/3.7	**82.2**/1.5/12.5/3.8	0.351
**Rank**				**Species**	**Species**	**Species**	**Species**
Tribe *Poeae*	*rbc*L	18/82	391 (2,5,15)	**23.8**/76.2	**11.5**/11.3/73.6/3.6	**11.5**/11.3/73.6/3.6	0.151
	*mat*K	18/79	354 (1,4,15)	**29.1**/70.9	**11.0**/28.8/52.0/8.2	**11.0**/28.8/52.0/8.2	0.100
	ITS	18/82	383 (2,5,15)	**44.6**/55.4	**32.4**/18.5/46.2/2.9	**24.0**/3.4/69.7/2.9	0.601
	*rbc*L+*mat*K	18/83	395 (2,5,15)	**31.4**/68.6	**15.2**/27.3/52.9/4.6	**4.2**/9.9/84.7/1.2	0.165
	*rbc*L+*mat*K+ITS	18/83	399 (2,5,15)	**39.3**/60.7	**26.1**/37.3/23.8/12.8	**20.6**/15.5/51.1/12.8	0.150
Briza	*rbc*L	1/2	7 (3,4,4)	**100**/0	**100**/0/0/0	**100**/0/0/0	0.100*
	*mat*K	1/2	4 (1,2,3)	**75**/25	**75**/12.5/0/12.5	**75**/0/0/25	0.100*
	ITS	1/2	7 (3,4,4)	**100**/0	**100**/0/0/0	**100**/0/0/0	0.150
	*rbc*L+*mat*K	1/2	7 (3,4,4)	**100**/0	**100**/0/0/0	**100**/0/0/0	0.100*
	*rbc*L+*mat*K+ITS	1/2	7 (3,4,4)	**100**/0	**100**/0/0/0	**100**/0/0/0	0.085
Catapodium	*rbc*L	1/2	6 (3,3,3)	**100**/0	**0**/16.7/83.3/0	**0**/0/100/0	0.100*
	*mat*K	1/2	5 (2,2,3)	**20**/80	**20**/20/60/0	**0**/0/100/0	0.100*
	ITS	1/2	5 (2,2,3)	**100**/0	**80**/0/0/20	**80**/0/0/20	0.160
	*rbc*L+*mat*K	1/2	6 (3,3,3)	**16.7**/83.3	**16.7**/33.3/50/0	**16.7**/16.6/66.7/0	0.100*
	*rbc*L+*mat*K+ITS	1/2	6 (3,3,3)	**50.0**/50.0	**59**/33.3/0/16.7	**50**/16.7/16.6/16.6	0.115
Cynosurus	*rbc*L	1/2	6 (3,3,3)	**33.3/66.7**	**33.3**/50.0/16.7/0	**100**/0/0/0	0.100*
	*mat*K	1/2	5 (2,2,3)	**100**/0	**80**/0/0/20	**80**/0/0/20	0.100*
	ITS	1/2	5 (2,2,3)	**100**/0	**100**/0/0/0	**100**/0/0/0	0.150
	*rbc*L+*mat*K	1/2	6 (3,3,3)	**83.3**/16.7	**83.3**/0/0/16.7	**83.3**/0/0/16.7	0.185
	*rbc*L+*mat*K+ITS	1/2	6 (3,3,3)	**100**/00	**83.3**/0/0/16.7	**83.3**/0/0/16.7	0.185
Hookerochloa	*rbc*L	1/2	5(2,2,3)	**100**/0	**80**/0/0/20	**80**/0/0/20	0.180
	*mat*K	1/2	5(2,2,3)	**40/60**	**33.4**/33.3/33.3/0	**0**/0/100/0	0.100*
	ITS	1/2	7(3,4,4)	**100**/0	**100**/0/0/0	**100**/0/0/0	0.160
	*rbc*L+*mat*K	1/2	6(3,3,3)	**50/50**	**33.3**/0/0/66.7	**33.3**/0/0/66.7	0.160
	*rbc*L+*mat*K+ITS	1/2	7(3,4,4)	**100**/0	**85.7**/0/0/14.3	**85.7**/0/0/14.3	0.170
Festuca	*rbc*L	1(9)	**26** (1/3/4)	**25.0**/75.0	**0.0**/7.1/82.2/10.7	**0.0**/7.1/82.2/10.7	0.100*
	*mat*K	1(9)	**23** (1/3/4)	**48.0**/52.0	**16.0**/32.0/44.0/8.0	**16.0**/32.0/44.0/8.0	0.100*
	ITS	1(9)	**25** (1/3/4)	**40.7**/59.3	**18.5**/14.8/33.4/33.3	**18.5**/3.7/44.4/33.3	0.245
	*rbc*L+*mat*K	1(9)	**26** (1/3/4)	**46.4**/53.6	**10.7**/7.1/67.9/14.3	**10.7**/7.1/67.9/14.3	0.100*
	*rbc*L+*mat*K+ITS	1(9)	**26** (1/3/4)	**60.7**/39.3	**53.6**/21.4/0.0/25.0	**35.7**/10.7/28.6/25.0	0.165

Taxon or clade	Genetic marker	Number of genera/species	Number of individuals<br><br>**[total (**minimum, **mean**, maximum)**]**	Specimen identification (%)			Threshold applied (% divergence)[a]
				NN<br>(**true**/false)	BCM<br>**(correct**/incorrect/ ambiguous/no identification**)**	TID<br>(**correct**/incorrect/ ambiguous/no identification)	
Lolium	*rbc*L	1(5)	**17** (1/4/9)	**38.9/**61.1	**0.0/**27.8/66.7/5.5	**0.0/**27.8/66.7/5.5	0.100*
	*mat*K	1(5)	**17** (1/4/9)	**27.8/**72.2	**0.0/**22.2/66.7/11.1	**0.0/**5.6/83.3/11.1	0.155
	ITS	1(5)	**16** (1/3/7)	**41.2/**58.8	**5.9/**29.4/52.9/11.8	**5.9/**17.7/64.7/11.7	0.245
	*rbc*L+*mat*K	1(5)	**18** (1/4/9)	**31.6/**68.4	**5.3/**21.0/57.9/15.8	**0.0/**21.0/63.2/15.8	0.080
	*rbc*L+*mat*K+ITS	1(5)	**19** (1/4/9)	**35.0/**65.0	**5.0/**45.0/40.0/10.0	**5.0/**15.0/70.0/10.0	0.155
Fine-leaved clade	*rbc*L	3/9	**28** (2,4,7)	**33.3/**66.7	**6.7/**6.6/66.7/20.0	**6.7/**6.6/66.7/20.0	0.180
	*mat*K	3/8	**22** (2,3,4)	**68.2/**31.8	**31.8/**22.7/18.2/27.3	**22.7/**0.0/50.0/27.3	0.245
	ITS	3/9	**26** (2,3,5)	**71.4/**28.6	**39.3/**7.1/14.3/39.3	**39.3/**3.6/17.8/38.3	0.245
	*rbc*L+*mat*K	3/10	**28** (2,3,5)	**46.7/**53.3	**10.0/**6.6/56.7/26.7	**10.0/**6.6/56.7/26.7	0.100*
	*rbc*L+*mat*K+ITS	3/10	**29** (2,3,5)	**67.7/**32.3	**45.2/**12.9/0.0/41.9	**45.2/**6.6/6.5/41.9	0.165
Parapholis	*rbc*L	1/2	**9** (3,4,6)	**66.7/**33.3	**11.1/**0/88.9/0	**11.1/**0/88.9/0	0.190
	*mat*K	1/2	**8** (3.4.5)	**50.0/**50.0	**50/**50/0/0	**0/**0/75/25	0.135
	ITS	1/2	**8** (2,4,6)	**87.5/**12.5	**87.5/**0/0/12.5	**87.5/**0/0/12.5	0.155
	*rbc*L+*mat*K	1/2	**9** (3,4,6)	**66.7/**33.3	**33.4/**33.3/22.2/11.1	**33.4/**33.3/22.2/11.1	0.075
	*rbc*L+*mat*K+ITS	1/2	**9** (3,4,6)	**55.6/**44.4	**44.5/**22.2/0/33.3	**44.5/**22.2/0/33.3	0.245
Poa	*rbc*L	1/48	**254** (2,5,15)	**10.2/**89.8	**3.9/**12.6/80.7/2.8	**3.9/**12.6/80.7/2.8	0.180
	*mat*K	1/47	**240** (2,5,15)	**19.2/**80.8	**6.7/**32.1/58.3/2.9	**6.7/**13.8/76.7/2.9	0.165
	ITS	1/48	**252** (2,5,15)	**28.6/**71.4	**10.3/**19.1/59.5/11.1	**10.3/**19.1/59.5/11.1	0.145
	*rbc*L+*mat*K	1/48	**256** (2,5,15)	**21.9/**78.1	**6.6/**30.5/57.8/5.1	**6.6/**30.5/57.8/5.1	0.075
	*rbc*L+*mat*K+ITS	1/48	**257** (2,5,15)	**26.5/**73.5	**14.4/**38.1/30.0/17.5	**11.2/**23.7/47.5/17.5	0.055
Puccinellia	*rbc*L	1/5	**16** (2,3,4)	**0/**100	**0/**11.1/88.9/0	**0/**11.1/88.9/0	0.100*
	*mat*K	1/4	**13** (3,3,4)	**56.3/**43.7	**25.0/**43.8/32.2/0.0	**12.5/**25.0/62.5/0.0	0.135
	ITS	1/5	**15** (2,3,4)	**58.8/**41.2	**29.4/**11.8/35.3/23.5	**29.4/**11.8/35.3/23.5	0.180
	*rbc*L+*mat*K	1/5	**16** (2,3,4)	**16.7/**83.3	**0/**22.2/72.2/5.6	**0/**22.2/72.2/5.6	0.100*
	*rbc*L+*mat*K+ITS	1/5	**16** (2,3,4)	**55.6/**44.4	**16.7/**5.6/22.2/55.6	**16.7/**5.6/22.2/55.6	0.235
Vulpia	*rbc*L	1(5)	**11** (1/2/5)	**75.0/**25.0	**16.7/**16.7/58.3/8.3	**16.7/**16.7/58.3/8.3	0.180
	*mat*K	1(6)	**5** (1/2/3)	**83.3/**16.7	**33.3/**0.0/0.0/66.7	**33.3/**0.0/0.0/66.7	0.245
	ITS	1(5)	**10** (1/2/5)	**50.0/**50.0	**25.0/**16.7/16.7/41.6	**16.7/**16.7/25.0/41.6	0.145
	*rbc*L+*mat*K	1(5)	**11** (1/2/5)	**58.3/**41.7	**0.0/**16.7/58.3/25.0	**0.0/**16.7/58.3/25.0	0.100*
	*rbc*L+*mat*K+ITS	1(5)	**12** (1/3/5)	**76.9/**23.1	**38.5/**0.0/0.0/61.5	**38.5/**0.0/0.0/61.5	0.080
Broad-leaved clade	*rbc*L	2/6	**27** (2,4,9)	**51.9/**48/1	**18.5/**14.8/63.0/3.7	**18.5/**14.8/63.0/3.7	0.180
	*mat*K	2/6	**26** (2,4,8)	**42.3/**57.7	**11.5/**23.1/65.4/0.0	**11.5/**23.1/65.4/0.0	0.135
	ITS	2/6	**25** (3,4,7)	**38.5/**61.5	**11.5/**23.1/46.2/19.2	**7.7/**11.5/61.5/19.2	0.155
	*rbc*L+*mat*K	2/6	**28** (2,4,9)	**42.9/**57.1	**10.7/**28.6/57.1/3.6	**10.7/**14.3/71.4/3.6	0.100*
	*rbc*L+*mat*K+ITS	2/6	**29** (3,4,9)	**55.2/**44.8	**24.1/**31.0/34.5/10.4	**13.8/**10.4/65.5/10.3	0.200
