# Supplementary material for: Testing efficacy of distance and tree-based methods for DNA barcoding of grasses (Poaceae tribe Poeae) in Australia
Source: PLoS One. 2017 Oct 30;12(10):e0186259. doi: 10.1371/journal.pone.0186259 (PMC5662090; doi:10.1371/journal.pone.0186259)
Supplement: S4 Table — BA, Bayesian inference; ITS, Internal transcribed spacer; ML, Maximum likelihood. aSuccess rates for generic determinations are indicated in bold. * Taxon represented by a single individual (singleton). (PDF) [file pone.0186259.s004.pdf]

S4 Table.

Recognized taxa (morphology)	Number of individuals (ITS)	Specimen identification (ITS)	Specimen identification (ITS)	Number of individuals (*rbc*L+*mat*K +ITS)	Specimen identification (*rbc*L+*mat*K +ITS)	Specimen identification (*rbc*L+*mat*K +ITS)
	**393**	**ML** correct/ ambiguous/ incorrect	**BA** correct/ ambiguous/ incorrect	**406**	**ML** correct/ ambiguous/ incorrect	**BA** correct/ ambiguous/ incorrect
Briza	11	**11/0/0** [a]	**11/0/0**	11	**11/0/0**	**11/0/0**
B. maxima	3	3/0/0	3/0/0	3	3/0/0	3/0/0
B. minor	4	4/0/0	4/0/0	4	4/0/0	4/0/0
B. subaristata	4	4/0/0	4/0/0	4	4/0/0	4/0/0
Catapodium	5	**5/0/0**	**2/3/0**	6	**5/1/0**	**0/6/0**
C. marinum	2	0/0/2	0/2/0	3	2/1/0	0/3/0
C. rigidum	3	2/1/0	2/1/0	3	3/0/0	0/3/0
Cynosurus	5	**5/0/0**	**5/0/0**	6	**3/3/0**	**5/1/0**
C. cristatum	2	2/0/0	2/0/0	3	0/3/0	2/1/0
C. echinatum	3	3/0/0	3/0/0	3	3/0/0	3/0/0
Dactylis	3	**3/0/0**	**3/0/0**	3	**3/0/0**	**3/0/0**
D. glomeratus	3	3/0/0	3/0/0	3	3/0/0	3/0/0
Dryopoa	4	**4/0/0**	**4/0/0**	5	**5/0/0**	**5/0/0**
D. dives	4	4/0/0	4/0/0	5	5/0/0	5/0/0
Festuca	27	**25/2/0**	**26/1/0**	28	**27/1/0**	**25/3/0**
F. arundinaceae	4	2/2/0	0/4/0	4	4/0/0	2/2/0
F. asperula	3	1/3/0	2/2/0	4	2/1/1	2/2/0
F. benthamiana	4	4/0/0	4/0/0	4	4/0/0	4/0/0
F. gautieri	1	0/1/0*	0/1/0*	1	0/1/0*	0/1/0*
F. muelleri	3	3/0/0	3/0/0	3	3/0/0	3/0/0
F. nigrescens	1	0/1/0*	0/1/0*	1	0/1/0*	0/1/0*
F. plebeia	4	0/3/1	0/4/0	4	0/3/1	0/4/0
F. pratensis	3	0/3/0	0/3/0	3	2/1/0	0/2/1
F. rubra	3	2/1/0	2/0/1	4	0/3/1	0/4/0
Hainardia	3	**0/3/0**	**0/3/0**	3	**2/0/1**	**0/3/0**
H. cylindrica	3	0/3/0	0/3/0	3	2/0/1	0/3/0
Hookerochloa	7	**7/0/0**	**7/0/0**	7	**7/0/0**	**7/0/0**
H. eriopoda	3	3/0/0	3/0/0	3	3/0/0	3/0/0
H. hookeriana	4	4/0/0	4/0/0	4	4/0/0	4/0/0
Lamarckia	3	**3/0/0**	**3/0/0**	3	**3/0/0**	**3/0/0**
L. aurea	3	3/0/0	3/0/0	3	3/0/0	3/0/0
Lolium	17	**17/0/0**	**17/0/0**	19	**19/0/0**	**19/0/0**
L. loliaceum	3	0/2/1	0/1/2	2	0/2/0	0/0/2
L. multiflorum	3	0/3/1	0/3/1	3	0/3/0	0/3/0
L. perenne	7	0/4/3	0/6/1	9	0/8/1	0/8/1
L. rigidum	3	0/2/1	0/3/0	4	0/4/0	0/4/1
L. temulentum	1	0/1/0*	0/1/0*	1	0/1/0*	0/1/0*
Parapholis	8	**7/0/1**	**3/5/0**	9	**8/0/1**	**0/9/0**
P. incurva	6	4/1/1	0/5/1	6	0/5/1	0/6/0
P. strigosa	2	2/0/0	0/2/0	3	0/3/0	0/3/0
Poa	255	**255/0/0**	**255/0/0**	260	**260/0/0**	**260/0/0**
P. affinis	2	0/2/0	2/0/0	2	0/2/0	2/0/0
P. amplexicaulis	4	0/4/0	0/4/0	4	0/4/0	0/4/0
P. annua	3	3/0/0	3/0/0	5	4/0/1	2/2/1
P. billardierei	5	5/0/0	5/0/0	5	2/3/0	5/0/0
P. bulbosa	6	6/0/0	6/0/0	6	6/0/0	6/0/0
P. cheelii	2	0/2/0	0/2/0	2	0/2/0	0/2/0
P. clelandii	6	0/6/0	0/6/0	6	0/6/0	0/6/0
P. clivicola	4	0/3/1	0/4/0	5	0/4/1	2/3/0
P. compressa	1	0/1/0*	0/1/0*	1	0/1/0*	0/1/0*
P. cookii	4	3/0/1	3/0/1	4	3/0/1	3/0/1
P. costiniana	6	0/6/0	0/6/0	6	0/6/0	0/6/0
P. crassicaudex	4	0/4/0	0/4/0	4	0/4/0	0/4/0
P. drummondiana	6	2/4/0	2/4/0	6	2/4/0	4/2/0
P. ensiformis	6	0/6/0	0/6/0	6	0/6/0	2/4/0
P. fawcettiae	6	0/6/0	0/6/0	6	0/6/0	0/6/0

Recognized taxa (morphology)	Number of individuals (ITS)	Specimen identification (ITS)	Specimen identification (ITS)	Number of individuals (*rbc*L+*mat*K +ITS)	Specimen identification (*rbc*L+*mat*K +ITS)	Specimen identification (*rbc*L+*mat*K +ITS)
	**393**	**ML** correct/ ambiguous/ incorrect	**BA** correct/ ambiguous/ incorrect	**406**	**ML** correct/ ambiguous/ incorrect	**BA** correct/ ambiguous/ incorrect
Poa fax	5	0/4/1	0/4/1	5	2/3/0	2/2/1
P. foliosa	8	7/0/1	7/0/1	8	7/0/1	7/1/0
P. fordeana	5	0/4/1	0/5/0	5	0/4/1	0/4/1
P. gunnii	6	0/6/0	0/6/0	6	0/6/0	0/6/0
P. halmaturina	1	0/1/0*	0/1/0*	1	0/1/0*	0/1/0*
P. hamiltoni	1	0/1/0*	0/1/0*	1	0/1/0*	0/1/0*
P. helmsii	5	0/5/0	0/5/0	5	0/5/0	0/5/0
P. hiemata	6	0/6/0	0/6/0	6	0/6/0	0/6/0
P. homomalla	4	0/4/0	0/4/0	4	0/4/0	0/4/0
P. hookeri	5	0/5/0	0/5/0	5	0/4/1	0/4/1
P. hothamensis	9	0/9/0	0/9/0	10	0/10/0	0/10/0
P. induta	7	0/7/0	0/7/0	7	0/6/1	0/6/1
P. infirma	4	4/0/0	4/0/0	4	2/2/0	0/4/0
P. jugicola	5	0/5/0	0/5/0	5	0/5/0	0/5/0
P. labillardierei	15	0/15/0	0/15/0	15	0/15/0	0/14/1
P. litorosa	3	0/3/0	0/3/0	3	0/3/0	0/3/0
P. lowanensis	5	0/5/0	0/5/0	5	0/5/0	0/5/0
P. meionectes	5	0/4/1	0/4/1	5	0/5/0	0/4/1
P. morrisii	6	0/6/0	0/6/0	6	0/6/0	0/6/0
P. mollis	4	0/4/0	0/4/0	4	0/4/0	0/4/0
P. orba	2	0/2/0	0/2/0	2	0/2/0	0/2/0
P. orthoclada	6	0/6/0	0/6/0	6	0/6/0	0/6/0
P. petrophila	3	0/3/0	0/3/0	3	0/3/0	0/2/1
P. phillipsiana	6	0/6/0	0/6/0	6	0/6/0	0/6/0
P. physoclina	4	0/4/0	0/4/0	4	0/4/0	2/0/2
P. poiformis	11	2/8/1	0/10/1	11	2/9/0	0/11/0
P. porphyroclados	6	2/4/0	2/4/0	6	2/4/0	2/4/0
P. pratensis	5	2/2/1	0/5/0	5	0/5/0	3/2/0
P. rodwayi	4	0/4/0	0/4/0	4	0/4/0	0/4/0
P. sallacustris	4	0/4/0	0/4/0	4	0/4/0	0/4/0
P. serpentum	2	0/2/0	0/2/0	2	0/2/0	0/2/0
P. sieberiana	15	0/14/1	0/14/1	15	2/13/0	0/15/0
P. tenera	7	0/7/0	0/6/1	7	0/7/0	0/6/1
P. trivialis	4	4/0/0	4/0/0	4	4/0/0	4/0/0
P. umbricola	2	0/2/0	0/2/0	2	0/2/0	0/2/0
Psilurus	3	**3/0/0**	**3/0/0**	3	**3/0/0**	**3/0/0**
P. incurvus	3	3/0/0	3/0/0	3	3/0/0	3/0/0
Puccinellia	17	**17/0/0**	**17/0/0**	18	**18/0/0**	**18/0/0**
P. ciliata	3	0/3/0	2/1/0	3	2/0/1	0/3/0
P. distans	2	2/0/0	2/0/0	2	2/0/0	0/2/0
P. fasciculata	3	3/0/0	3/0/0	3	3/0/0	3/0/0
P. longior	1	0/1/0*	0/1/0*	1	0/1/0*	0/1/0*
P. perlaxa	4	3/0/1	0/4/0	4	2/0/2	0/4/0
P. stricta	3	0/3/0	0/3/0	4	0/3/1	0/4/0
P. vassica	1	0/1/0*	0/1/0*	1	0/1/0*	0/1/0*
Saxipoa	3	**0/3/0**	**0/3/0**	3	**0/3/0**	**0/3/0**
S. saxicola	3	0/3/0	0/3/0	3	0/3/0	0/3/0
Sclerochloa	5	**5/0/0**	**5/0/0**	5	**5/0/0**	**5/0/0**
S. dura	5	5/0/0	5/0/0	5	5/0/0	5/0/0
Sphenopus	3	**3/0/0**	**3/0/0**	3	**3/0/0**	**3/0/0**
S. divaricatus	3	3/0/0	3/0/0	3	3/0/0	3/0/0
Sylvipoa	2	**2/0/0**	**2/0/0**	2	**2/0/0**	**2/0/0**
S. queenslandica	2	2/0/0	2/0/0	2	2/0/0	2/0/0
Vulpia	10	**10/2/0**	**10/2/0**	12	**10/2/0**	**10/2/0**
V. bromoides	3	2/0/1	0/3/0	3	0/3/0	0/3/0
V. ciliata	1	0/1/0*	0/1/0*	1	0/1/0*	0/1/0*

Recognized taxa (morphology)	Number of individuals (ITS)	Specimen identification (ITS)	Specimen identification (ITS)	Number of individuals (*rbc*L+*mat*K +ITS)	Specimen identification (*rbc*L+*mat*K +ITS)	Specimen identification (*rbc*L+*mat*K +ITS)
	**393**	**ML** correct/ ambiguous/ incorrect	**BA** correct/ ambiguous/ incorrect	**406**	**ML** correct/ ambiguous/ incorrect	**BA** correct/ ambiguous/ incorrect
Vulpia fasciculata	2	2/0/0	2/0/0	2	2/0/0	2/0/0
V. muralis	1	0/1/0*	0/1/0*	1	0/0/1	0/0/1
V. myuros	5	0/5/0	0/5/0	5	0/5/0	0/5/0
**Rates for Genera**						
Number		**383/7/3**	**379/12/2**		**394/7/5**	**363/40/3**
Percentage		97.4/1.8/0.8	96.4/3.1/0.5		97.1/1.7/1.2	89.4/9.9/0.7
**Rates for Species**						
Number		**112/256/25**	**103/274/16**		**127/259/20**	**104/282/20**
Percentage		28.5/65.1/6.4	26.2/69.7/4.1		31.3/63.8/4.9	25.6/69.5/4.9
